# Tilted *vs.* parallel assembly caused birefringent reversal in columnar phases of oligothiophene as well as applications for LEDs and hole transport materials

**DOI:** 10.1039/d5sc04039f

**Published:** 2025-08-14

**Authors:** Shibo Chen, Xuyang Du, Qingqing Han, Jingjing Luo, Fen Wang, Jiaming Liu, Yang Yu, Xiaohong Cheng

**Affiliations:** a Key Laboratory of Medicinal Chemistry for Natural Resource, Ministry of Education, Yunnan Research & Development Center for Natural Products, School of Chemical Science and Technology, Yunnan University Kunming 650091 P.R. China xhcheng@ynu.edu.cn; b School of Materials and Energy, Yunnan University Kunming 650091 P. R. China yuyang@ynu.edu.cn

## Abstract

Polycatenar columnar liquid crystals *n*T (*n* = 1–6) of π-conjugated oligothiophenes end-capped with bulky *N*-tris(hexadecyloxy)benzyl carbazole groups exhibit unusual molecular tilted and parallel assemblies, realizing the reversal of birefringence within the columnar liquid crystalline (LC) phases depending on core length and temperature. In the parallel arrangement of short core compounds 2T and 3T, molecules align perpendicular to the column axis. The stacking of these molecules involves a rotational component, resulting in local helical assembly, and this specific packing motif collectively stabilizes the Col_hex_/*p*6*mm* phase with negative birefringence. In the tilted arrangement of long core compounds 5T and 6T, the long rigid core-driven tilted assemblies induce a structural transition to the Col_rec_/*c*2*mm* phase, accompanied by a characteristic reversal of birefringence from negative to positive. In particular, compound 4T forms a Col_hex_/*p*6*mm* phase at lower temperature, but transforms into a Col_rec_/*c*2*mm* phase at higher temperature, accompanied by an inversion of optical birefringence. These special LC nanostructures provide important insights into the correlation between the molecular structure and assembly orientation in π-conjugated oligothiophenes. Furthermore, with an increase in π-conjugation length, these compounds exhibit bright blue-to-red emission in both solution and thin-film states, covering the entire visible spectrum and enabling their use in the preparation of full-color light-emitting diodes (LEDs), including white light. Most interestingly, solution-processable nano-films derived from columnar polycatenar oligothiophenes exhibit excellent hole mobility. Thus, this work proposes a strategy to develop polycatenar LC semiconductors that exhibit full-color emission.

## Abstract 2

The unusual molecular tilt and parallel assemblies in oligothiophene liquid crystals achieve reversal of optical birefringence. These materials are also used in full-color light-emitting diodes and hole transport materials.

## Graphical abstract

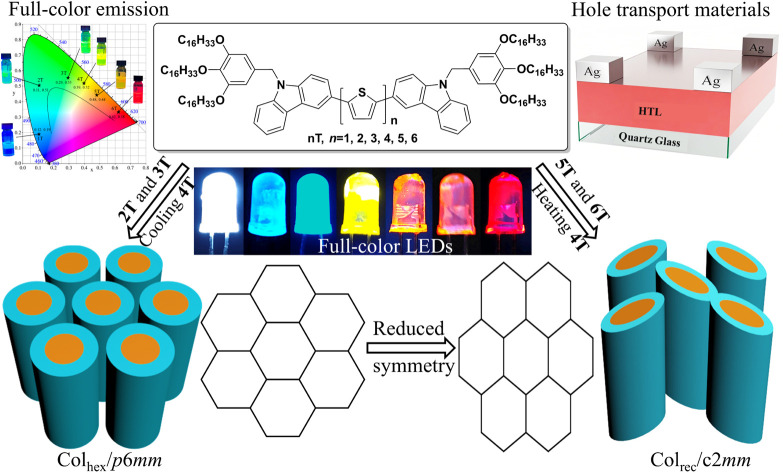

## 1. Introduction

The controllable self-assembly of π-conjugated building blocks into ordered supramolecular nanostructures has gained significant momentum, enabling targeted modulation of optoelectronic properties for organic semiconductors.^1^ The introduction of liquid crystallinity for π-conjugated substances represents a promising approach to modulate molecular self-organization processes, thereby improving their dynamic and anisotropic characteristics.^2^ Among different functionalized soft materials, columnar liquid crystals have garnered considerable attention due to their capacity to create highly ordered one-dimensional (1D) superstructures, which are useful for charge and ion transport,^3^ molecular separation,^4^ catalysis,^5^ and photonic device applications.^6^ In the past few years, π-conjugated polycatenar compounds, including naphthalene diimides,^7^ diketopyrrolopyrrole,^8^ and [1]benzothieno [3,2-*b*]benzothiophene,^9^ have been established as columnar liquid crystalline (LC) semiconductors. The innovative design of novel π-conjugated molecules and the development of nanomaterials with specific configurations are essential for creating columnar liquid crystals with integrated photoelectric properties. In this regard, we have recently demonstrated that several linearly extended π-conjugated compounds can self-assemble into LC nanostructures.^10^ The electronic performance of such materials and the operational efficiency of resultant devices fundamentally depend on precise control of their self-assembled nanostructures and macroscopic alignment.^11^

Oligothiophenes represent a prominent class of organic semiconductors,^12^ and their derivatives have found widespread applications as field-effect transistors (FETs),^13^ hole-transport materials (HTMs)^14^ and organic light-emitting diodes (OLEDs).^15^ Substantial research efforts have been directed toward engineering oligothiophene derivatives with tailored molecular geometries, enabling the formation of highly ordered molecular assemblies through self-assembly processes.^16^ Although many oligothiophene-based LC compounds have been reported, the mesophases observed in these systems remain predominantly limited to smectic or nematic phases, with only a few examples demonstrating columnar LC phases.^17^ This structural constraint primarily arises from their rod-like molecular geometric characteristics. As an aromatic heterocyclic compound consisting of two fused benzene rings and a pyrrole ring, carbazole has been widely used as a fundamental component of organic semiconductors, particularly in the development of light-emitting devices.^18^ The photophysical and electrochemical properties of carbazole derivatives, including photoluminescence, phosphorescence, electroluminescence, and charge transport, have been extensively investigated.^19^ Carbazole also serves as a key component in the development of luminescent LC materials. Moreover, carbazole-based liquid crystals have enabled the realization of nematic,^20^ smectic^21^ and columnar phases,^22^ and their potential in OLEDs has been explored. In our previous work, two bulky carbazole terminal groups were introduced at both ends of a dimeric thiophene core (DT),^23^ achieving not only a hexagonal columnar mesophase but also excellent green luminescence. However, the self-assembly behaviors and further practical applications (particularly in organic semiconductors) of carbazole-capped oligothiophene-based compounds with longer π-conjugated lengths remain unexplored. Therefore, we aim to combine the unique redox, electrical, and optical properties of highly π-conjugated carbazole-capped oligothiophenes with the dynamic LC self-assembly behavior.

**Scheme 1:**
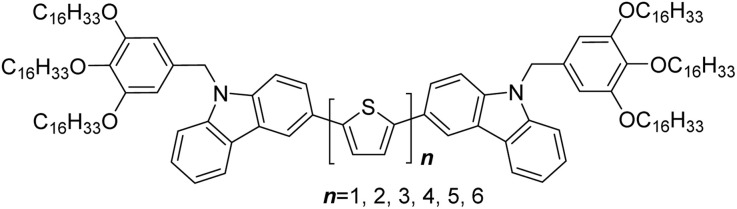
Molecular structure of *n*T.

Herein, a series of novel carbazole-capped oligothiophene polycatenar liquid crystals *n*T (*n* = 1–6) was designed and synthesized, in which different numbers of thiophene units were employed as a rigid core and bulky *N*-tris(hexadecyloxy)benzyl carbazole groups were capped at both terminals (Scheme 1). The self-assembly behavior, photoluminescence, redox and semiconducting properties of these π-conjugated polycatenar oligothiophenes were investigated. These molecules exhibit hybrid geometric structures that integrate rod-like LC motifs and disc-like molecular features, exhibiting unusual molecular tilted and parallel assemblies as well as realizing the reversal of birefringence. With the adjustment of the π-conjugated length, these compounds achieve full-color luminescence with high quantum yields. Furthermore, detailed SEM and AFM studies show that the machinability of these compounds enables the preparation of defect-free, smooth films without pinholes, resulting in high hole mobility. This approach provides a straightforward and effective technique for adjusting the self-assembly characteristics and functionalities of π-conjugated LC materials.

## 2. Results and discussion

### 2.1 Synthesis

**Scheme 2:**
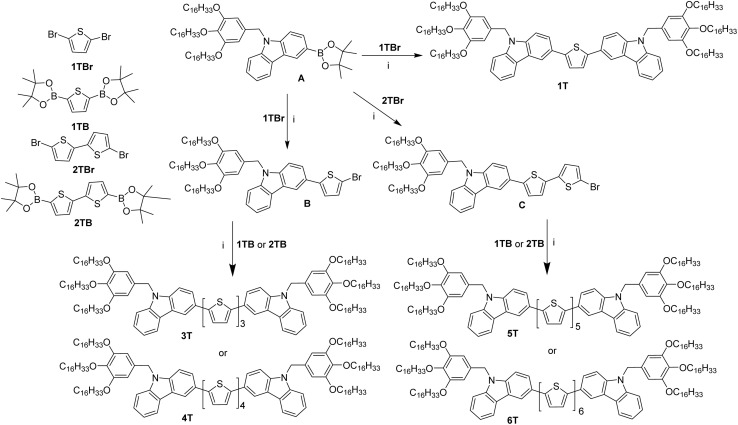
Synthesis of *n*T. Reagents and conditions: Pd(PPh_3_)_4_, K_2_CO_3_, H_2_O, THF, N_2_, 80 °C, 12 h.

A series of π-conjugated polycatenar oligothiophenes *n*T with different numbers of thiophene units was designed and synthesized (Scheme 2). We have previously reported the synthesis of compounds 3,4,5-tris(hexadecyloxy)benzyl-9*H*-carbazole-3-boronic acid bis(pinacol)ester (A) and 2T.^23^ The synthesis of compound 1T was achieved by the double Suzuki cross-coupling of 2,5-dibromothiophene (1TBr) with two equivalents of A in the presence of Pd(PPh_3_)_4_ as a catalyst. Subsequently, under standard Suzuki coupling conditions, compound A reacted with one equivalent of 1TBr or 5,5′-dibromo-2,2′-bithiophene (2TBr) to give compounds B or C, respectively. Two equivalents of compound B were further cross-coupled with 2,5-bis-thiopheneboronic acid pinacol ester (1TB) or 2,2′-bithiophene-5,5′-diboronic acid bis (pinacol) ester (2TB) to yield 3T or 4T. Similarly, compound C underwent a Suzuki coupling reaction with 1TB or 2TB to generate 5T or 6T (see the SI for details).

### 2.2 LC properties and structures

Polarized optical microscopy (POM), differential scanning calorimetry (DSC), and small-angle and wide-angle X-ray scattering (SAXS and WAXS) were utilized to examine the LC characteristics of *n*T. The thermal phase behaviors are summarized in Table 1. The results indicated that, except for 1T, all polycatenar oligothiophenes can form stable columnar LC phases. Notably, 2T, 3T, 5T and 6T exhibit single mesophase behavior, whereas 4T shows bismesophases during heating and cooling cycles.

**Table 1:** LC properties and XRD data of *n*T^a^

Comp.	*T*/°C [Δ*H*/J g^−1^]	*a*/nm (*T*/°C)	*μ*
1T	H: Cr 99^b^ iso	—	—
	C: Iso 96^b^ Cr		
2T	H: Cr 74.9 [13.4] Col_hex_/*p*6*mm* 118.1 [1.6] I	*a*_hex_ = 5.30 (110)	3.0 (*p*6*mm*)
	C: I 115.1 [1.7] Col_hex_/*p*6*mm* 62.6 [15.9] Cr		
3T	H: Cr 81.9 [14.2] Col_hex_/*p*6*mm* 122.9 [7.9] I	*a*_hex_ = 5.59 (90)	3.2 (*p*6*mm*)
	C: I 92.0 [1.2] Col_hex_/*p*6*mm* 73.5 [1.7] Cr		
4T	H: Cr 56.8 [11.8] Col_hex_/*p*6*mm* 97.7 [2.5] Col_rec_/*c*2*mm* 165.4 [2.9] I	*a*_hex_ = 6.00 (80), *a*_rec_ = 7.12 (150), *b*_rec_ = 4.72	3.0 (*p*6*mm*), 1.8 (*c*2*mm*)
	C: I 155.2 [2.6] Col_rec_/*c*2*mm* 89.4 [2.5] Col_hex_/*p*6*mm* 48.1 [7.8] Cr		
5T	H: Cr 56.5 [35.6] Col_rec_/*c*2*mm* 168.2 [0.4] I	*a*_rec_ = 7.10 (100), *b*_rec_ = 5.12	1.9 (*c*2*mm*)
	C: 160.8 [0.9] Col_rec_/*c*2*mm* 46.6 [33.6] Cr		
6T	H: Cr 53.6 [22.6] Col_rec_/*c*2*mm* 210.6 [0.2] I	*a*_rec_ = 7.62 (100), *b*_rec_ = 5.37	2.1 (*c*2*mm*)
	C: 208.9 [0.3] Col_rec_/*c*2*mm* 43.5 [23.1] Cr		

a Transitional temperatures were determined by DSC (peak temperature, second H (heating) and C (cooling) scan); abbreviations: Cr = crystal; I: isotropic liquid; Col_hex_/*p*6*mm*: hexagonal columnar phase; Col_rec_/*c*2*mm*: rectangular columnar phase with a *c*2*mm* lattice; *a*: lattice parameter measured by SAXS; *μ*: number of molecules in a columnar unit for Col_hex_/*p*6*mm*
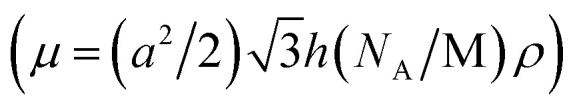
 and Col_rec_/*c*2*mm* phases (*μ* = (*ab*/2)*h* (*N*_A_/M)ρ), *ρ* = 1 g cm^−3^ (approximate value); *h* = the height of each stratum of the columns measured by WAXS.

b Determined by POM.

#### 2.2.1 Col_hex_/*p*6*mm* LC phase of short core compounds

**Fig. 1:**
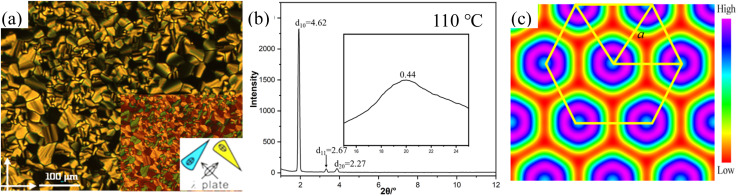
(a) The optical texture of 2T at 110 °C; the inset shows the textures with an additional λ-retarder plate. (b) SAXS pattern of the Col_hex_/*p*6*mm* phase of 2T at specific temperatures; the inset shows the corresponding WAXS pattern. (c) Reconstructed ED map of the Col_hex_/*p*6*mm* phase showing a hexagonal plane with aromatic cores and alkyl chains.

The typical spherulitic textures were observed under POM on cooling the isotropic liquids of 2T and 3T between crossed polarizers, indicating columnar phases. The coexistence of the dark and birefringent spherulite regions in the same sample is characteristic of a typical optical uniaxial columnar phase (Fig. 1a and S1a), which can have a hexagonal or square lattice. The texture of the columnar phase shows negative birefringence (inset in Fig. 1a), indicating that the primary π-conjugated pathway is oriented perpendicular to the long axis of the column. X-ray diffraction (XRD) studies of 2T and 3T reveal the *d*-spacings of distinct reflections within the small-angle region (Fig. 1b), aligning in the ratio of 
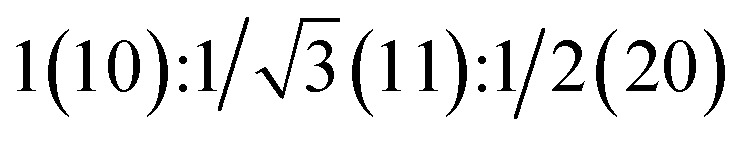
. This supports the presence of a hexagonal lattice of *p*6*mm* symmetry, with lattice parameters *a*_hex_ = 5.30 and 5.59 nm, respectively. The characteristics of dispersion observed in WAXS (Fig. 1b) indicate the absence of fixed molecular positions, thereby affirming the highly dynamic behavior of the LC phase. Using these data, we can determine the molecular number (*μ*) arranged in a given column length (*h*). Although *h* can be chosen freely, we assume that for 2T and 3T, the values for the Col_hex_/*p*6*mm* phase are 0.44 nm and 0.42 nm, which are results aligned with the broad signal observed in the XRD patterns. Selecting these particular values does not necessarily imply that 0.44 nm or 0.42 nm represents a precise columnar repetition distance, but it suggests the presence of periodicity at this distance, and the signal's width indicates that this correlation is maintained across multiple molecules. In this manner, the molecular numbers in the *h* column height are both calculated to be approximately 3 (trimer) for the Col_hex_/*p*6*mm* phase (Table 1). The electron density (ED) map reconstructed from SAXS diffraction intensities further reveals a simple hexagonal columnar plane. The *p*6*mm* lattice is marked by high electron density areas (blue to purple), which signify the locations of the columns of conjugated units, whereas the aliphatic side chains are found in the low-density areas (yellow to red) (Fig. 1c).

**Fig. 2:**
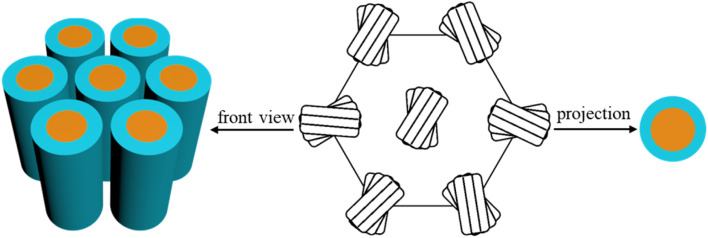
Molecular stacking model of the Col_hex_/*p*6*mm* phase.

For the mesophases of 2T and 3T, although the calculated maximum extended molecular length (*L*_max_ = 7.2 nm for 2T, Fig. S4a) exceeds the lattice parameter *a*_hex_ (5.30 nm for 2T), the molecular conformation is not fully extended due to cross-linking and folding of alkyl chains, resulting in an effective molecular length close to *a*_hex_. Additionally, the intramolecular π-conjugated route aligns parallel to the *p*6*mm* lattice, as evidenced by the optically negative fan-shaped texture (Fig. 1a). Considering the Col_hex_/*p*6*mm* phase and the associated symmetry criteria, the column should possess a circular cross-section that is oriented perpendicularly to its axis. However, the trimer formed by three molecules tends to be elliptical rather than circular. Nevertheless, the trimers are capable of free rotation about the column axis while maintaining perpendicular orientation relative to it. Projection of the assemblies onto a plane perpendicular to the column axis further results in a circular cross-section (Fig. 2).^24^ It is worth noting that we did not observe the characteristic *Fddd* LC phase with long-range helical order in 2T and 3T.^10*d*^ Therefore, in the Col_hex_/*p*6*mm* phase, the rotation of the trimers can be left- or right-handed, which unavoidably leads to numerous helical inversions. Energy calculation according to the quadrupolar attraction–repulsion theory shows that to avoid steric hindrance between alkyl chains, the mutual orientation of trimers in adjacent columns will incline to approach verticality as much as possible.^10*d*^ Consequently, in the columnar phase, the columns display an average circular cross-section along their axes and maintain their positions within the typically favored hexagonal lattice, owing to the locally related helix of the trimers. Furthermore, the formation of the Col_hex_/*p*6*mm* phase can be explained by acknowledging the symmetry of its hexagonal lattice, which effectively achieves space filling. If the chain segments are fluid enough and exhibit liquid-like behavior under these conditions, the system is likely to favor a hexagonal arrangement with high symmetry. Consequently, it is suggested that the columnar structure is influenced by the symmetries present in the mesophase, along with the orientation of the flexible chains and the π-conjugated core. The molecular dynamics (MD) model of Materials Studio was used to further confirm the molecular stacking process of the Col_hex_/*p*6*mm* phase (Fig. S5a). The trimers turn and arrange themselves along the column axis to occupy the available space within the columns. The nanosegregation of the rigid core and the surrounding alkyl chains, combined with the vertical intermolecular π–π stacking, jointly promotes the molecular assembly into columnar nanostructures.

#### 2.2.2 Col_rec_/*c*2*mm* LC phase of long core compounds

**Fig. 3:**
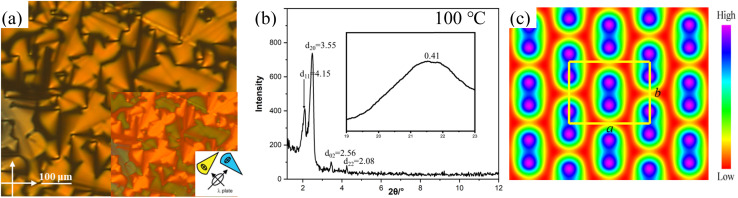
(a) The optical texture of 5T at 100 °C; the inset shows the texture with an additional λ-retarder plate. (b) SAXS pattern of the Col_rec_/*c*2*mm* phase of 5T at specific temperatures; the inset shows the corresponding WAXS pattern. (c) Reconstructed ED map of the Col_rec_/*c*2*mm* phase showing a rectangular plane with aromatic cores and alkyl chains.

Before discussing the temperature-dependent phase transition of compound 4T, we should first discuss the Col_rec_/*c*2*mm* phase of long-core compounds 5T and 6T. The fan-shaped textures of columnar phase with surprising positive birefringence were observed under POM on cooling the isotropic liquids of 5T and 6T between crossed polarizers (Fig. 3a and S1b). The emergence of optical biaxiality in columnar phases implies a reduction in the symmetry of the assembly macrostructure.^25^ Namely, with extension of the π-conjugation, both 5T and 6T exhibit new LC phases. In the SAXS pattern of 5T, two strong and sharp peaks appear at 4.15 nm and 3.55 nm, accompanied by two distinct diffraction peaks at 2.56 nm and 2.08 nm (Fig. 3b). Indexing of these peaks to the (11), (20), (02), and (22) diffraction planes confirmed a Col_rec_/*c*2*mm* phase with lattice parameters *a*_rec_ = 7.10 nm and *b*_rec_ = 5.12 nm. Notably, 6T adopts the comparable LC phase with lattice parameters *a*_rec_ = 7.62 nm and *b*_rec_ = 5.37 nm (Fig. S3b). The molecular numbers are both calculated to be approximately 2 (dimer) for the Col_rec_/*c*2*mm* phase (Table 1). The ED map of the rectangular columnar phase confirms a *c*2*mm* lattice for both 5T and 6T (Fig. 3c).

**Fig. 4:**
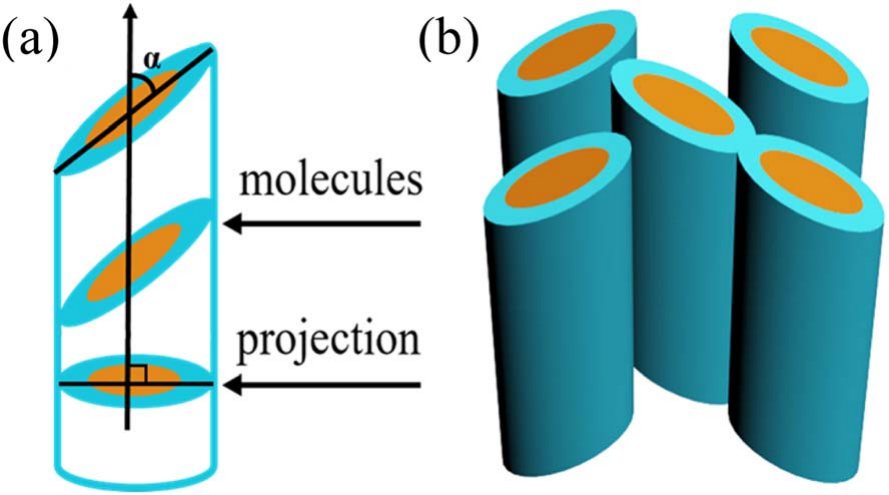
Molecular tilted stacking model of the Col_rec_/*c*2*mm* phase.

Compared to 2T and 3T, the molecular shapes of 5T and 6T are more inclined to an elliptical form. The molecular lengths of 5T and 6T measured along their maximum extension are 8.2 nm and 8.5 nm, respectively (Fig. S4d and S4e). It is worth noting that these lengths are significantly greater than the distance between columns in the Col_rec_/*c*2*mm* phase (*a*_rec_/2). Therefore, it is proposed that the elliptical molecular dimers of 5T and 6T are arranged in an inclined manner within the columns (Fig. 4). This kind of tilted stacking is reported to be suitable for molecules with similar geometries.^26^ Observations under POM with a λ-plate confirmed that within the LC range, 5T and 6T, compared to 2T and 3T, undergo a reversal of the birefringence signal from negative to positive. This reversal indicates that the tilt angle of the dimer in the Col_rec_/*c*2*mm* phase crosses the magic angle (54.7°) relative to the columnar axis.^27^ Based on longitudinal offset, under ideal conditions of the fully extended molecular geometry, without alkyl chain interdigitation, the tilt stacking angles (*α* = 90° − cos^−1^*a*_rec_/2*L*_max_) of the molecules in the columns relative to the column axis are estimated to be 26° (5T) and 27° (6T). The MD model confirms the rationality of tilted stacking (Fig. S5b).

#### 2.2.3 LC properties of compound 4T

**Fig. 5:**
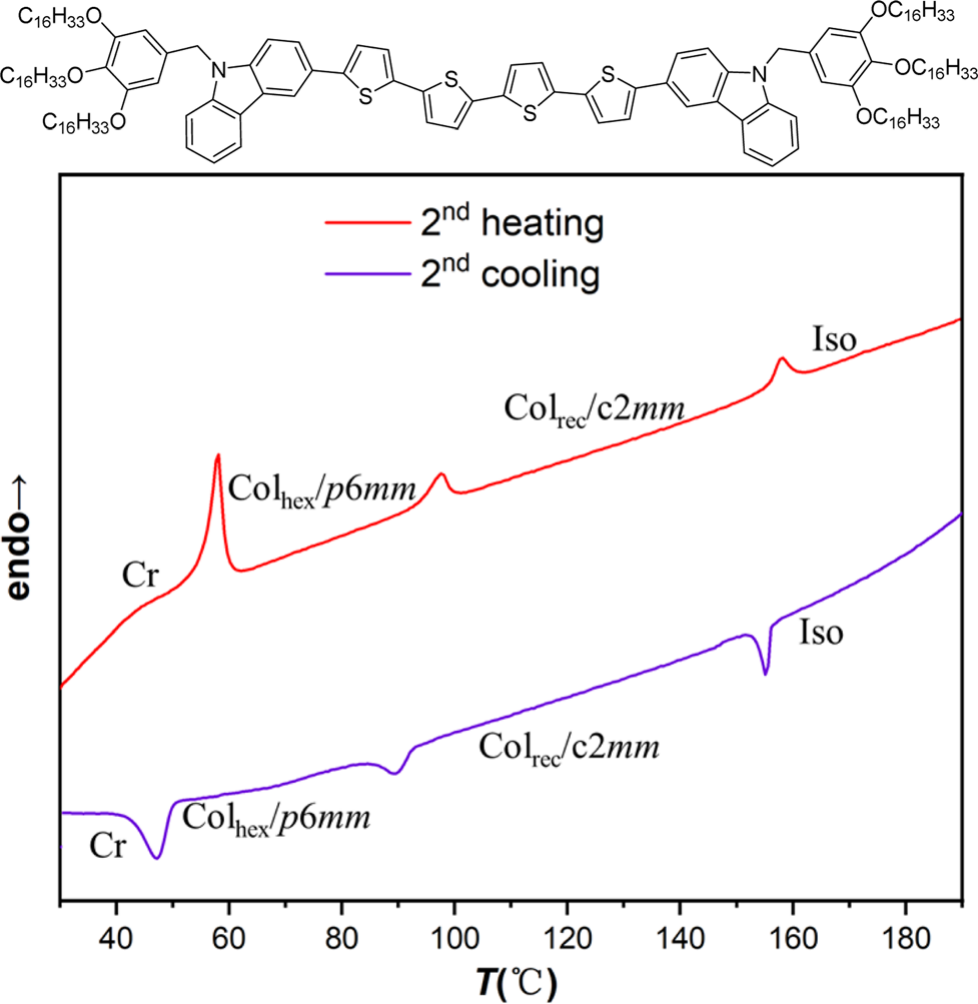
Chemical structure of compound 4T and its DSC traces.

**Fig. 6:**
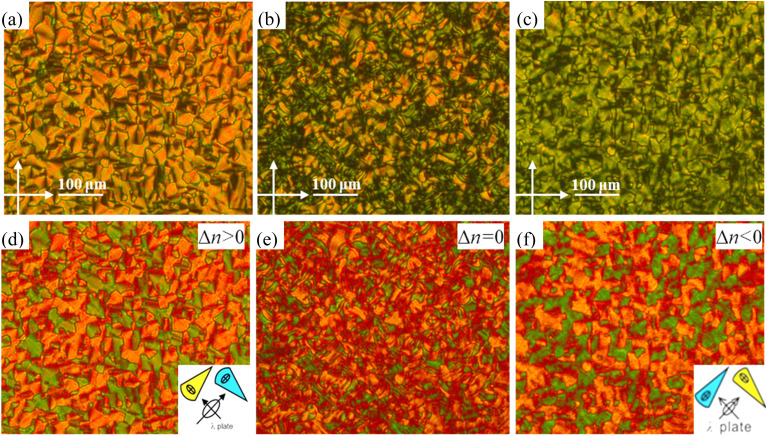
(a–c) The optical textures of 4T are shown between crossed polarizers when it cools from Col_rec_ (155 °C) *via* a transient phase (89 °C) to the Col_hex_/*p*6*mm* phase (87 °C); the textures with an additional λ-retarder plate are shown in (d–f).

**Fig. 7:**
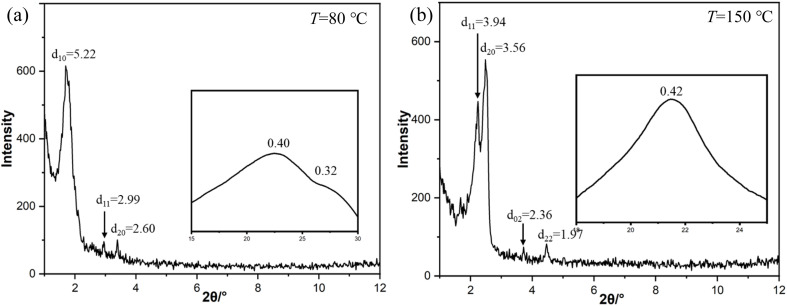
SAXS patterns of Col_hex_/*p*6*mm* (a) and Col_rec_/*c*2*mm* (b) phases of 4T at specific temperatures. The insets show the corresponding WAXS patterns.

In the cooling process of compound 4T from isotropic liquid, three phase transition peaks were detected at 155 °C, 89 °C and 48 °C by DSC (Fig. 5). Within the temperature range (155–48 °C), WAXS exhibits a diffuse scattering pattern, characteristic of a LC phase lacking fixed molecular positional order (insets in Fig. 7). Characteristic fan-shaped textures were observed at the first transition (155–89 °C), indicating a columnar LC phase (Fig. 6a). The positive birefringence of the mesophase demonstrates optical biaxiality (Fig. 6d). During the phase transition of 4T at 89 °C, this birefringent texture becomes optically chaotic (Fig. 6b and e). Subsequently, investigation using an additional λ-plate reveals an inversion in the orientation of yellow and blue fan patterns at the 89–48 °C transition (Fig. 6f). This observation indicates an inversion in the birefringence sign from positive to negative at lower temperatures. At this stage, the coexistence of dark regions and negative birefringence fan-shaped textures within the same sample is characteristic of a hexagonal or square lattice (Fig. 6c).

The XRD pattern of 4T measured in the low-temperature range is shown in Fig. 7a. There are three distinct peaks in the small-angle area (5.22 nm, 2.99 nm and 2.60 nm) in the SAXS pattern of 4T. The observed diffraction spacing ratio of 
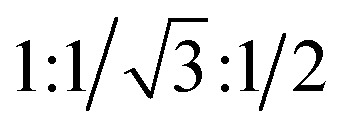
 (indexed as (10), (11) and (20) reflections) confirms the Col_hex_/*p*6*mm* LC phase with lattice parameter *a*_hex_ = 6.00 nm, as shown in Fig. 7a. Alongside the LC diffusion halo centered at 0.40 nm in the WAXS region, a broad shoulder observed at 0.32 nm corresponds to the characteristic π–π stacking distance within the columnar structure, suggesting strong core–core interactions resulting from the enhanced planarity of the 4T molecular core.^28^ Upon heating, the Col_hex_/*p*6*mm* phase of 4T changed to a Col_rec_/*c*2*mm* phase, as evidenced by two strong and sharp peaks at 3.94 nm and 3.56 nm in the high-temperature SAXS pattern, along with two obvious diffraction peaks at 2.36 nm and 1.97 nm (Fig. 7b). The indices of the (11), (20), (02) and (22) diffraction planes confirmed the Col_rec_/*c*2*mm* phase with lattice parameters *a*_rec_ = 7.12 nm and *b*_rec_ = 4.73 nm. The molecular numbers of 4T are calculated to be approximately 3 for the Col_hex_/*p*6*mm* phase and 2 for the Col_rec_/*c*2*mm* phase (Table 1).

Just as in the assembly processes of short-core compounds 2T and 3T, alkyl chain cross-linking and folding make the effective molecular length close to *a*_hex_ for the low-temperature mesophase of 4T. Therefore, in the hexagonal columnar phase of 4T, trimers can freely rotate around the column axis to form locally correlated helical twists while remaining perpendicular to the column axis. The columns display an averaged circular cross-section along their axes and maintain their positions in the favorable hexagonal lattice. In this Col_rec_/*c*2*mm* phase of 4T, the intercolumnar distances are significantly shorter than the molecular length (*L*_max_ = 7.8 nm, Fig. S4c), indicating pronounced molecular tilting within the columns.^28^ The observed phenomenon coincides with a negative-to-positive birefringence inversion at the phase transition between the Col_hex_/*p*6*mm* and Col_rec_/*c*2*mm* mesophases (Fig. 6d–f). Based on the maximally extended length of 4T in the rectangular columnar phase, the tilt angle is estimated to be approximately 27° at *T* = 150 °C. This molecular tilt assembly may confer thermodynamic advantages by providing an entropic gain, which facilitates enhanced fluctuation of the conjugated core along the column axis.^29^ Thus, during the Col_hex_/*p*6*mm*-Col_rec_/*c*2*mm* transition, periodic band textures are observed under POM (Fig. 6). This behavior likely originates from fluctuations in columnar order coupled with variations in the tilt angle within the tilted molecular stacking of the columns. Significantly, the two large sterically hindered benzyloxy groups at the carbazole location would marginally impede the formation of the rectangular columnar mesophase. However, the diffuse halo, which suggests a significant core–core interaction (equivalent to the 0.32 nm peak of the 4T Col_hex_/*p*6*mm* phase), was the only peak seen in the wide-angle region of the XRD pattern.^28^ In other words, intermolecular core–core interactions facilitate displacement between adjacent π-systems, thereby promoting the emergence of core tilting within the rectangular columnar phase.

**Fig. 8:**
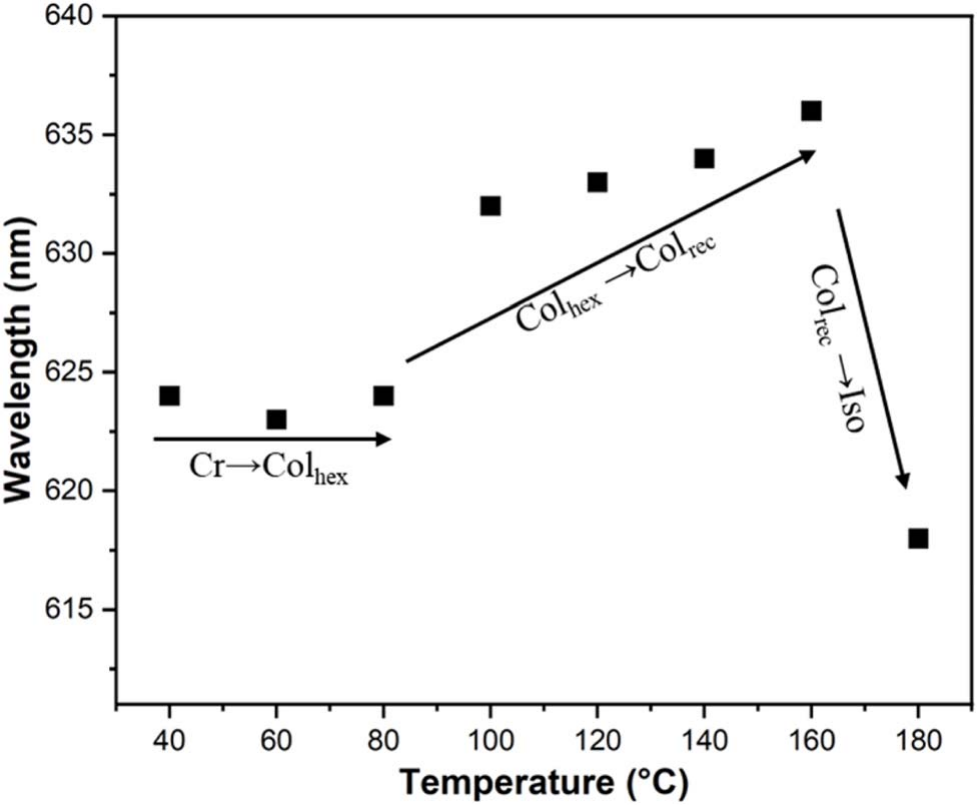
Photoluminescence spectrum behavior of 4T depending on the temperature during the heating process.

Fig. 8 shows the change in the maximum emission wavelength of 4T as a function of temperature during heating. The transition between the crystalline state and the Col_hex_/*p*6*mm* phase does not show significant changes. In the two-phase transition intervals of 40–80 °C and 100–160 °C, the Col_rec_/*c*2*mm* phase (632 nm) exhibits a slight red shift compared with the Col_hex_/*p*6*mm* phase (624 nm). The unusual photoluminescent characteristics of the Col_rec_/*c*2*mm* phase are probably attributed to the slanted orientation of the stacked core in relation to the columnar axis.^30^ Based on the exciton coupling theory, molecular sliding-induced J-aggregation emerges as the angle of the molecular plane relative to the aggregate alignment is less than 54.7°.^31^ Although bending and folding of the alkyl chains shorten the effective molecular length, resulting in a tilt angle of less than 27° (relative to the maximum theoretical value of the column axis) in the Col_rec_/*c*2*mm* phase, it is still below 54.7°. Therefore, the temperature-dependent variation in the emission spectrum is ascribed to the inclined arrangement of the cores in the Col_rec_/*c*2*mm* phase.

The most critical aspect of the Col_hex_/*p*6*mm*–Col_rec_/*c*2*mm* transition of 4T lies in its second-order feature, characterized by the absence of discontinuous changes in cross-sectional area during the transformation from a circular (Col_hex_/*p*6*mm*: *S* = π*a*_hex_^2^/4 = 28 nm^2^) to an elliptical (Col_rec_/*c*2*mm*: *S* = π*a*_rec_*b*_rec_/4 = 26 nm^2^) geometry. This stands in stark contrast to liquid crystals with polycatenar characteristics reported earlier, in which the same transition shows both a significant enthalpy change (suggestive of pronounced first-order behavior) and a sudden alteration in cross-sectional area.^32^

**Fig. 9:**
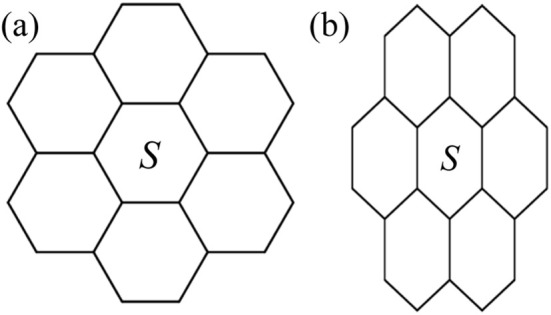
The columnar hexagonal net transitions from the regular *p*6*mm* symmetry (a) to the less symmetric *c*2*mm* lattice (b) while maintaining an almost constant cross-sectional area *S*.

Since the Col_hex_/*p*6*mm*–Col_rec_/*c*2*mm* phase transition occurs upon increasing the core length or on heating the sample, it may be driven by directional enhancement of molecular rigidity rather than isotropic motion. On the one hand, an increase in the number of thiophenes significantly enhances the rigidity of the molecular structure. On the other hand, the strong tendency of sufficiently long hexadecyl chains to align in parallel at high temperatures reduces their fluidity, thereby increasing molecular rigidity. However, the transformations of the LC phase do not necessarily induce changes in the cross-sectional area of the lattice. This interpretation can be framed as follows. While the analysis primarily addresses circular and elliptical cross-sectional areas, the geometric definition of *S* as the hexagonal unit area within the network in Fig. 9a is theoretically sound. Fig. 9b demonstrates the same network after deformation, where the hexagonal shape transitions from regular to irregular while preserving its original area. This simplified model describes the symmetry reduction from the *p*6*mm* to the *c*2*mm* space group. At the molecular level, this phenomenon arises from directional suppression of chain mobility.^24^ Consequently, it can be appropriately concluded that throughout this 4T transition, the diminution in chain mobility would result in a varying spatial distribution, ultimately leading to a phase characterized by distinct symmetry following the transition. This argument aligns with previous reports indicating that Col_hex_/*p*6*mm* phase formation is governed by chain mobility.^24^ Thus, as chain mobility decreases, the system loses its capacity to self-assemble into a circular cross-sectional arrangement, leading to symmetry degradation. The increase in molecular rigidity ultimately drives the phase transition, aligning with the observed second-order characteristics of the transition, since catastrophic reconfiguration of cross-sectional dimensions is not required. Thus, the discovered core length-dependent birefringence switching during LC phase transitions established a new paradigm for understanding the dynamic coupling mechanism among molecular conformation, order parameters, and optical responses in soft matter systems.

### 2.3 Full-color emission

In recent years, using π-conjugated systems to create materials with full-color luminescence has been intensively studied.^33^ A common strategy is to modify luminescent materials with different conjugated lengths to induce red or blue shifts in their emission spectra while maintaining a high photoluminescence quantum yield (*Φ*_PL_). To enhance material stability, thiophene units are often incorporated into the π-conjugated system by combining them with luminescent cores. Such materials have been widely applied in OLEDs,^34^ fluorescent probes^35^ and bioimaging.^36^ As a readily tunable chromophore, carbazole has demonstrated excellent luminescent properties in our prior research.^23^ Here, we precisely modulate the π-conjugated length by introducing varying numbers of thiophene units, thereby yielding high-efficiency luminescent materials that exhibit tunable full-color emission.

**Fig. 10:**
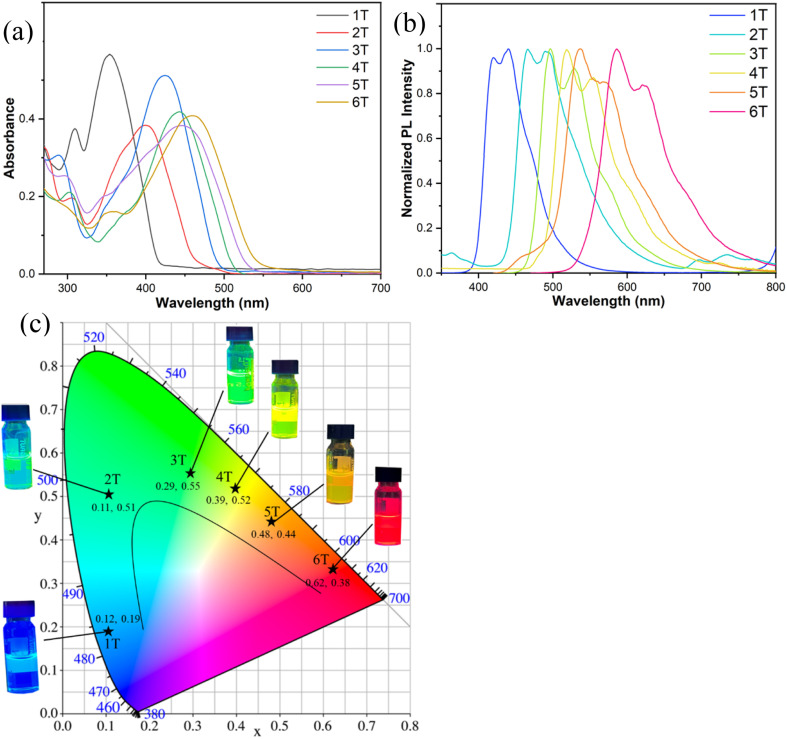
Spectral information of *n*T in DCM solution (10^−5^ M). (a) Absorption spectra; (b) PL spectra; (c) chromaticity coordinate chart of the six compounds. The insets show the photoluminescence images (365 nm) of six compounds in DCM.

The UV-vis absorption spectra of *n*T were investigated in dichloromethane (Fig. 10). These compounds exhibit a weak (around 300 nm) and a strong absorption peak (between 350 and 500 nm), with specific maxima at 355 nm (1T), 400 nm (2T), 425 nm (3T), 442 nm (4T), 450 nm (5T) and 462 nm (6T). These absorption features are primarily attributed to π–π* electronic transitions. Notably, the extension of π-conjugation leads to a substantial red shift in absorption peaks and significantly alters the absorption characteristics of these LC compounds.

When irradiated with a 365 nm UV lamp, the six compounds exhibited distinct emission colors of blue, cyan, green, yellow, orange and red in dichloromethane solutions, respectively (insets of Fig. 10c). Analysis of their emission spectra revealed maximum emission wavelengths at 440 nm (1T), 468 nm (2T), 496 nm (3T), 519 nm (4T), 539 nm (5T) and 587 nm (6T), corresponding to the observed chromatic progression (Fig. 10b). To quantitatively characterize the emission colors, we calculated the CIE chromaticity coordinates from their emission spectra. As shown in Fig. 10c, the obtained coordinates were (0.12, 0.19), (0.11, 0.51), (0.29, 0.55), (0.39, 0.52), (0.48, 0.44) and (0.62, 0.38) for compounds 1T–6T, respectively, confirming the visual observations. The emission spectra collectively span the entire visible spectrum from blue to red, with compound 6T additionally demonstrating near-infrared emission extending to 780 nm. This spectral evolution aligns with the absorption characteristics, showing a progressive red shift in emission wavelength with increasing π-conjugated length. The majority of these compounds exhibit strong emission intensity, as further evidenced by their measured *Φ*_PL_ (Table 2). Compounds 3T and 4T demonstrate particularly prominent luminescence, with *Φ*_PL_ values approaching 0.8 in dichloromethane solution. 1T, 2T, 5T and 6T also display bright emission, showing *Φ*_PL_ values of 0.45, 0.31, 0.39, and 0.52, respectively, under identical measurement conditions. In addition, the emission peak is divided into two maxima, indicating the occurrence of aggregation and excimer emission due to the combined intermolecular interactions involving carbazole and thiophene, which facilitate co-planarization of conjugated groups in the excited molecular state.^37^ This further enhances the π-conjugation effect and the planar core conformation of molecules, facilitating charge transfer.

**Table 2:** Summarization of photophysical properties of *n*T

Comp.	Solvents	*λ*_abs_ (nm)	*λ*_em_ a (nm)	*Φ*_PL_ b
1T	DCM	355	440	0.45
	Film	364	470	0.15
2T	DCM	400	468	0.31
	Film	407	507	0.21
3T	DCM	425	496	0.78
	Film	419	548	0.33
4T	DCM	442	519	0.80
	Film	452	625	0.39
5T	DCM	450	539	0.65
	Film	473	636	0.24
6T	DCM	462	587	0.52
	Film	486	688	0.17

a Excited at the absorption maxima peak.

b Solution: relative quantum yields (the calculation method is provided in the SI); film: absolute quantum yields.

**Fig. 11:**
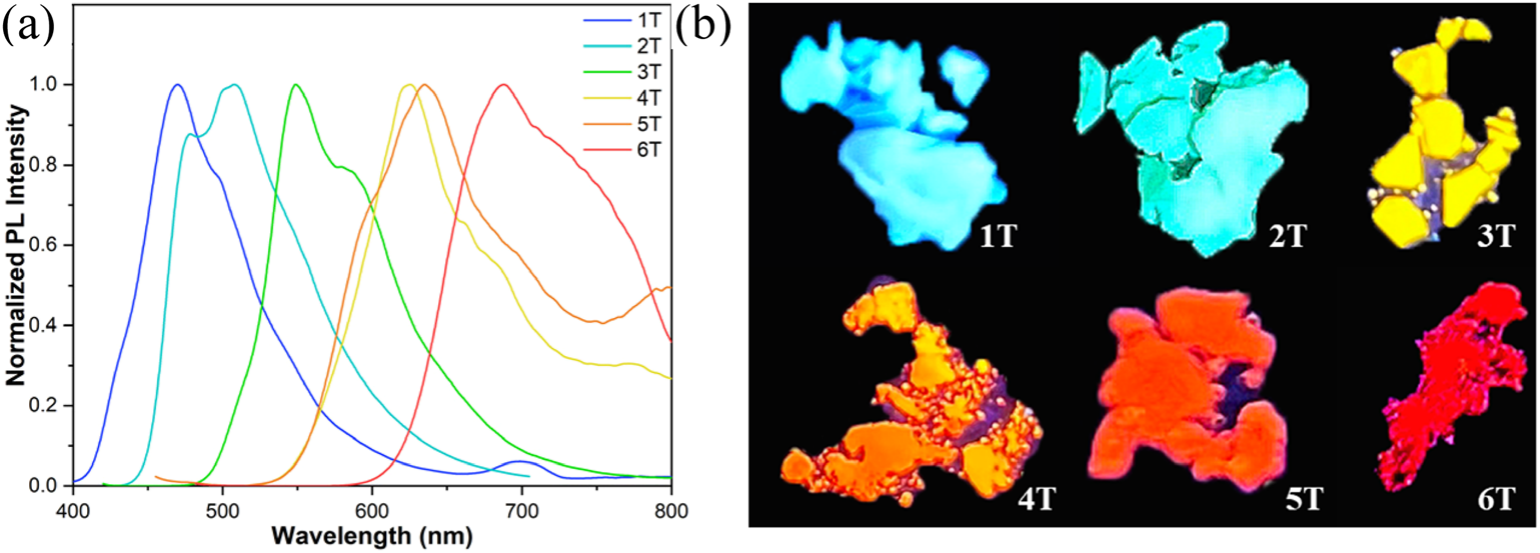
(a) Photoluminescence spectrum of *n*T in the film; (b) photoluminescence of *n*T powders under 365 nm UV lamp irradiation.

The compounds that exhibit luminescence in the aggregated state are more conducive to practical applications.^23^ All six derivatives were found to maintain favorable luminescent properties in the solid state, exhibiting emission colors spanning from blue to deep red in powdered form (Fig. 11b), thereby achieving full-color coverage across the visible spectrum (Fig. 11a). Compared to their solution-phase counterparts, the solid-state emission spectra of all compounds display significant red shifts (Δ*λ* = 30–99 nm) (Table 2), likely attributable to vibrational relaxation processes induced by intermolecular interactions.^38^ Although these compounds exhibit substantially lower *Φ*_PL_ in the solid state than in solution, they retain appreciable solid-state luminescence, with *Φ*_PL_ values ranging from 0.15 to 0.39 (Table 2). This combination of broad spectral tunability and retained emissive efficiency positions these compounds as promising candidates for developing full-color luminescent materials.

**Fig. 12:**
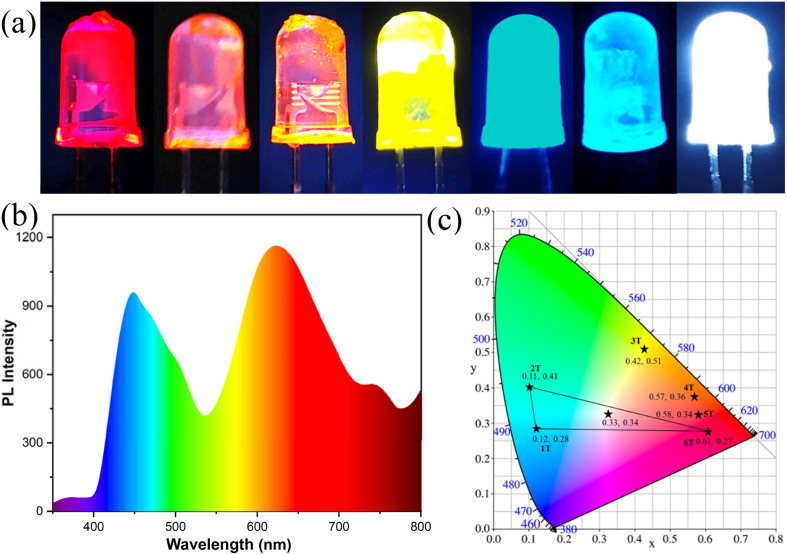
(a) The full-color LEDs displayed when 365 nm bulbs coated with *n*T films are electrified; (b) PL spectrum of WLEDs; (c) CIE of the full-color LEDs.

Based on the excellent luminescent properties of these compounds, they were used to fabricate full-color LEDs to demonstrate their light-emitting performance and validate their practical utility. We selected a dip-coating process to prepare the LEDs. First, 2 mg of *n*T were dissolved in 1 ml of dichloromethane, followed by the addition of 95 wt% polyethylene glycol to form a viscous mixture. The mixture was coated onto a 365 nm UV LED bulb, and the solvent was dried using a hair dryer. Upon electrification (3.2 V), these LEDs emitted bright light, achieving full-color LED functionality (Fig. 12a). For white light-emitting diode (WLED) fabrication, red/green/blue luminescent materials are required as color conversion layers. Compounds 1T, 2T, and 6T closely match these emission colors and thus were selected to prepare WLEDs using the aforementioned method (weight ratio: 1T : 2T : 6T = 1 : 0.8 : 0.6). The electrified WLED emitted bright white light with an emission spectrum covering the entire visible range (Fig. 12b), a chromaticity coordinate of (0.33, 0.34) within the white light region (Fig. 12c), and a luminous efficiency of 53 lm W^−1^. These devices, with their outstanding performance, demonstrate significant potential for commercial development.

### 2.4 Theoretical calculation and electrochemical properties

**Fig. 13:**
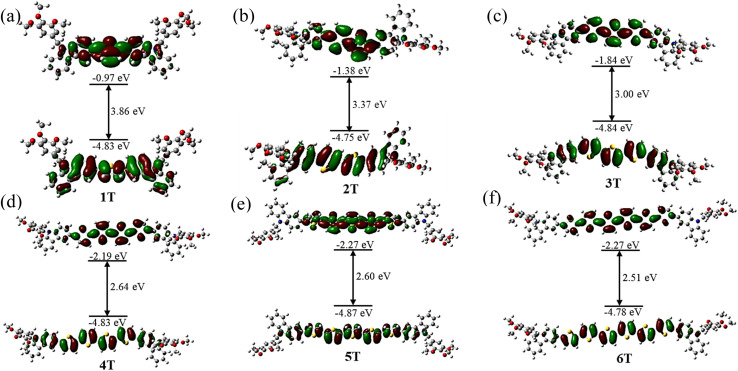
Electron density distribution and energy levels of the HOMO and LUMO for *n*T (the long alkyl chains have been replaced by methyl).

To study the characteristics of the ground state and low-lying singlet excited states of *n*T, as well as their association with molecular structure, density functional theory (DFT) calculations were conducted on *n*T utilizing the B3LYP/6-311G level of theory. As shown in Fig. 13, the HOMO–LUMO energy gap (Δ*E*_g_) for the six compounds are 3.86 eV (1T), 3.37 eV (2T), 3.00 eV (3T), 2.64 eV (4T), 2.60 eV (5T) and 2.51 eV (6T). This implies that the absorption or emission wavelengths associated with the HOMO–LUMO transition progressively redshift as the π-conjugation length increases. Notably, the energy level alignment of 4T–6T enables effective hole extraction toward the electrode, regardless of whether the holes are generated *via* photoexcitation or an applied voltage.^39^ This mechanism plays a critical role in achieving a higher open-circuit voltage (*V*_OC_) in solar cells.^40^ In addition, the HOMO and LUMO orbitals of 4T–6T exhibit delocalization across the molecular core, resulting in effective overlap and local excitation behavior.^37^ Thus, their charge transfer characteristics can be confirmed. By modulating geometric distortions linked to charge transfer, this property significantly lowers the reorganization energy (*λ*) of molecular assembly.^41^ Based on the semi-classical Marcus theory, a lower reorganization energy promotes excited-state geometric relaxation upon charge injection, thereby accelerating intermolecular charge hopping rates.^42^ Reorganization energies of the neutral molecules under positive charging were calculated with the four points method, using the Gaussian 09 program at the B3LYP/6-311G level.^43^ The B3LYP functional is known to give accurate geometries for similar π-conjugated molecules. The computed reorganization energies are shown in Fig. S8. The results show that 4T (161.47 meV), 5T (151.50 meV), and 6T (145.06 meV) exhibit lower reorganization energies (Table S7), which suggests that these compounds may possess higher carrier mobilities.^44^ Furthermore, comparison of the reorganization energies for compounds 1T to 6T reveals a gradual decrease with the extension of π-conjugation in the oligothiophenes. This trend reflects enhanced stability in larger π-conjugated systems, which is more favorable for charge transport.^45^

**Fig. 14:**
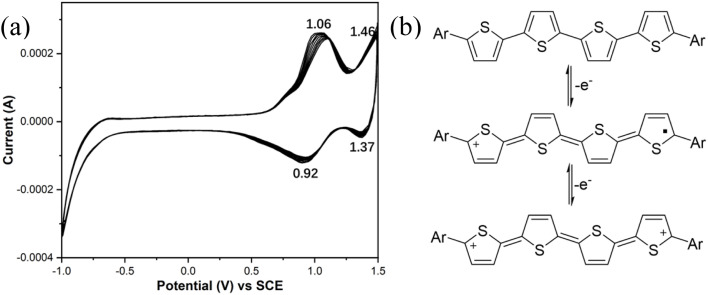
(a) Cyclic voltammogram of 4T in 0.1 M Bu_4_NPF_6_/acetonitrile electrolyte with a scan rate of 100 mV s^−1^ (cycle 10 times); (b) the chemical structure change of oligothiophene in the redox process.

Prior research has demonstrated that tetrathiophene derivatives exhibit favorable electrochemical characteristics, which are essential for the applications of LC compounds in optoelectronic materials. Considering that 4T–6T exhibit similar redox processes, the representative compound 4T was selected for studying electrochemical properties. The cyclic voltammogram of 4T in Bu_4_NPF_6_/acetonitrile electrolyte is shown in Fig. 14a. The data reveal two successive reversible one-electron oxidation events characterized by half-wave potentials (*E*_1/2_) of 0.99 and 1.42 V relative to the SCE (Saturated Calomel Electrode), corresponding to the stepwise generation of cationic radicals and the oligothiophene dication, respectively (see Fig. 14b).^17*e*^ The redox reactions demonstrate reproducibility (at least ten times) during repeated scans, since the extended π-conjugation of the oligothiophene framework enhances the stability of the oxidized species. Consequently, employing 4T–6T as materials for hole transport could lead to promising outcomes.

### 2.5 Charge transport properties

**Fig. 15:**
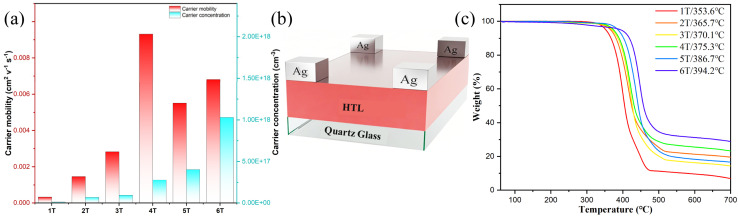
(a) Carrier mobility of *n*T HTLs; (b) Hall device structure; (c) TGA curves of 1T–6T. The measurements were performed under a nitrogen atmosphere, with heating rates of 10 °C min^−1^.

In the technology for preparing solution-processable organic materials, the presence of unwanted crystal defects frequently results in pore formation and dendritic growth within the film, which adversely impact charge transport by entrapment of charge carriers at the grain boundaries.^46^ Our purpose is to investigate the semiconductor characteristics of solution-processable films of polycatenar oligothiophenes. It is worth noting that previous studies on charge transport in polycatenar LC derivatives have focused on melt-processable samples,^47^ which are limited to the micron scale and have not been fully utilized in advanced technologies. Therefore, this study aims to bridge this gap by using a Hall measurement system to explore the electronic properties of polycatenar oligothiophenes in solution-processed films (Fig. 15a). By doing so, we can gain valuable insights into their charge carrier mobility and unlock their full potential in future technological advances. To accurately measure the carrier mobility, a carrier diode device was fabricated using a quartz glass/*n*T (100 nm)/Ag (100 nm) structure (Fig. 15b). The results indicate that 4T exhibits the highest carrier mobility, reaching 9.31 × 10^−3^ cm^2^ V^−1^ s^−1^. This value is significantly higher than those of the other hexagonally columnar phase compounds 2T (1.46 × 10^−3^ cm^2^ V^−1^ s^−1^) and 3T (2.83 × 10^−3^ cm^2^ V^−1^ s^−1^), as well as the crystalline compound 1T (3.32 × 10^−4^ cm^2^ V^−1^ s^−1^) with lowest conjugation. Therefore, factors such as the π-bridge conjugation length likely influence the carrier mobility of the HTMs. As mentioned, 4T possesses superior conjugation compared to 1T–3T HTMs, which contributes to its higher carrier mobility.^14*b*^ It is noteworthy that 4T exhibits higher carrier mobility than 5T (5.51 × 10^−3^ cm^2^ V^−1^ s^−1^) at room temperature, even though the latter has a greater conjugation length. One possible explanation for this observation is the enhanced molecular packing order within the columns of the Col_hex_/*p*6*mm* phase for 4T, which facilitates carrier hopping between the vertically aligned molecules. In contrast, the carrier mobilities of 5T and 6T (6.81 × 10^−3^ cm^2^ V^−1^ s^−1^) are higher than that of 3T, which may be attributed to their sufficiently long thiophene conjugation being adequate to overcome the detrimental effects associated with the tilted stacking in their rectangular columnar phase. The carrier mobility of 4T–6T is comparable to that of previously reported oligothiophene derivatives, which provides opportunities for the application of oligothiophene liquid crystal compounds in the semiconductor field.^48^

Further Hall effect measurements were performed to analyze the carrier concentration of *n*T as an HTM. The carrier concentration of 4T was measured to be 2.78 × 10^17^ cm^−3^. With the extension of π-conjugated length, the carrier concentrations of 5T and 6T were further increased, reaching optimal values of 4.02 × 10^17^ cm^−3^ and 2.03 × 10^18^ cm^−3^, respectively (Table 3). In contrast, compounds 1T–3T with lower conjugation degrees exhibit comparatively lower carrier concentrations. Subsequent Hall effect measurements confirmed that the *n*T thin film is a p-type semiconductor. In p-type semiconductors, where holes are the majority carriers, the carrier mobility closely approximates the hole mobility, and the hole concentration dictates the total carrier concentration.^49^ Therefore, 4T–6T exhibit promising potential as high-performance hole transport materials.

**Table 3:** The carrier transport properties of 1T–6T films^a^

HTMs	Carrier mobility (cm^2^ V^−1^ s^−1^)	Carrier concentration (cm^−3^)
1T	3.32 × 10^−4^	1.12 × 10^16^
2T	1.46 × 10^−3^	6.74 × 10^16^
3T	2.83 × 10^−3^	9.37 × 10^16^
4T	9.31 × 10^−3^	2.78 × 10^17^
5T	5.51 × 10^−3^	4.02 × 10^17^
6T	6.81 × 10^−3^	1.03 × 10^18^

a Carrier transport properties of *n*T were measured at room temperature.

The thermal stability of the compounds is a crucial parameter for evaluating their performance as HTMs. Fig. 15c shows the thermogravimetric analysis (TGA) data of 1T–6T. The results show decomposition temperatures of 353.6 °C for compound 1T, 365.7 °C for 2T, and 370.1 °C for 3T, while compounds 4T–6T all exhibit decomposition temperatures exceeding 375 °C. Therefore, all compounds demonstrate relatively high thermal stability, confirming their suitability as LC semiconductors.^14*b*^

**Fig. 16:**
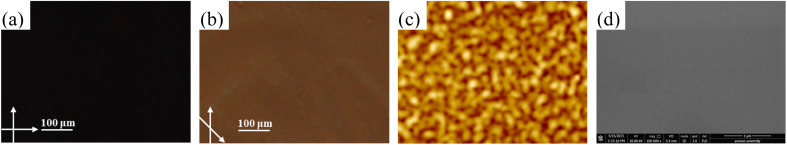
(a) POM image of 4T (100 nm thin film) at room temperature; arrows represent the relative direction of the polarizer and analyzer (polarizer and analyzer are 90° crossed); (b) POM image of the same slide (45°); (c) AFM image of the 4T thin film; (d) SEM image showing the continuity and conformity of 4T.

To further prove the uniformity of these dynamic LC materials, the surface of the films was studied by POM. The films showed a dark appearance under cross-polarizers (Fig. 16a), indicating ordered stacking perpendicular to the substrate. By adjusting the angle of the analyzer and polarizer to 45° (Fig. 16b), a uniform defect-free bright field was observed. Atomic force microscopy (AFM) was then used to examine the nanoscale surface characteristics of the films. The AFM images in Fig. 16c and S7 reveal detailed surface characteristics distinct from the bulk morphology, confirming the formation of pinhole-free smooth films. These findings are consistent with the SEM image of the 4T film shown in Fig. 16d. Given the excellent uniformity and order of the 4T–6T LC films, the material was spin-coated onto nanodevices for charge transport research to achieve optimal morphological characteristics.

**Fig. 17:**
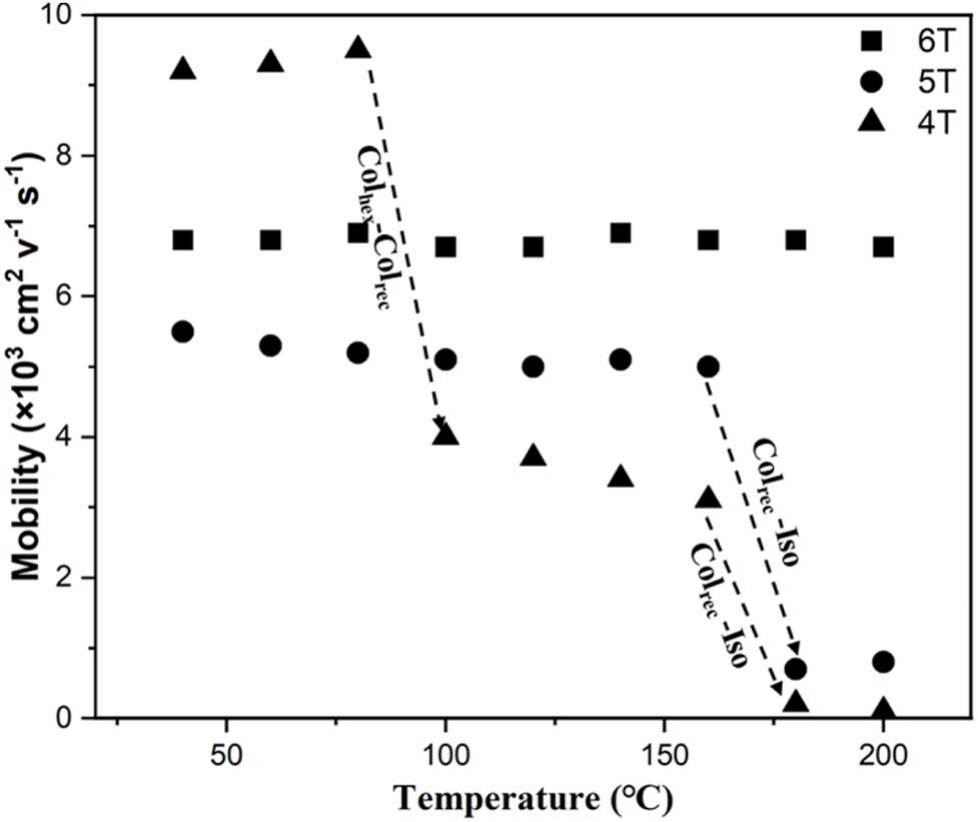
Temperature dependence of carrier mobility. The dashed lines denote the phase transitions.

The charge transport properties of oligothiophenes within specific temperature ranges were further investigated. Fig. 17 shows the results of mobility variations with temperature. Throughout the LC temperature range of the Col_rec_/*c*2*mm* phase, both 5T and 6T displayed temperature-independent charge transport properties, leading to almost constant carrier mobility values of roughly 5.5 × 10^−3^ and 6.8 × 10^−3^ cm^2^ V^−1^ s^−1^, respectively. These observations suggest that effective intra-columnar charge transport is attained in the Col_rec_/*c*2*mm* phase without significant perturbations from positional disorder or energy fluctuations.^50^ During the isotropic-Col_rec_/*c*2*mm* phase transition, the mobilities of 4T and 5T increased abruptly, indicating that the increased carrier mobility is caused by the tilted stacking arrangement of oligothiophenes in the Col_rec_/*c*2*mm* phase. Upon further cooling, 4T underwent a Col_rec_/*c*2*mm*–Col_hex_/*p*6*mm* phase transition, and its mobility sharply increased to 9.5 × 10^−3^ cm^2^ V^−1^ s^−1^ at 80 °C. The relatively high mobility can be attributed to the enhanced molecular packing ordering within the columns of the low-temperature Col_hex_/*p*6*mm* phase, which facilitates carrier hopping between vertically aligned molecules.^51^ Based on this experiment, it is revealed that the supramolecular structure of polycatenar oligothiophenes in columnar nanostructures is crucial to their enhanced electronic properties. The future work will explore the application of these oligothiophene-based polycatenar liquid crystals as HTLs in perovskite solar cells.

## 3. Conclusion

In summary, unlike conventional rod-like oligothiophene liquid crystals, the polycatenar molecules reported here, featuring bulky *N*-tris(hexadecyloxy)benzyl carbazole end groups and an oligothiophene core, exhibit a highly interesting and rare columnar phase transition (Col_hex_/*p*6*mm* ↔ Col_rec_/*c*2*mm*). Crucially, this architecture enables reversible switching of the birefringence sign (negative ↔ positive). The first is core-length-dependent inversion. Short cores (2T and 3T) adopt vertical stacking (negative birefringence), whereas long cores (5T and 6T) exhibit tilted stacking (positive birefringence). The second mechanism is temperature-driven inversion, specifically observed in 4T. This compound undergoes a rare thermal transition, which changes from negative birefringence in the Col_hex_ phase to positive birefringence in the Col_rec_ phase. This constitutes the first demonstration of dynamically controllable birefringence reversal in oligothiophene-based liquid crystals. Critically, the discovery of core-length-dependent and temperature-dependent birefringence switching during LC phase transitions establishes a novel paradigm for understanding tilted columnar assembly in soft matter systems. Furthermore, the vast majority of columnar LC phases exhibit negative birefringence due to the typical alignment where the high polarizability axis is perpendicular to the column axis. Consequently, columnar phases with positive birefringence are exceptionally rare and typically require specialized molecular designs to induce tilted or parallel alignment of the high polarizability axis. Therefore, the compounds *n*T establish a robust platform and provide a general design strategy for achieving positive birefringence in LC materials. Precise control over molecular assembly *via* tailored structures and thermal stimuli enables tunable material properties, making these nanoscale LC phases industrially significant for applications such as microelectronics fabrication.^52^

In addition, the compound family reported in this work achieves bright full-spectrum emission (blue to red) through precise thiophene-mediated conjugation length control. Crucially, this performance persists in both solution and solid states, which has rarely been investigated for LC compounds, particularly oligothiophene-based derivatives. Furthermore, these compounds act as efficient emissive layers in solution-processable LEDs, enabling bright full-color emission including white light. By eliminating the need for complex doping and multilayer architectures, this property significantly enhances the practical utility of oligothiophene liquid crystals for luminescent materials.

Most interestingly, these oligothiophene-based polycatenar liquid crystals uniquely combine columnar self-assembly with solution-treatable self-healing film formation to produce semiconductors with phase-controllable properties. Unlike films from traditional methods that yield micron-scale films with grain boundary defects, *n*T thin films exhibit large-area, defect-free ordered alignment suitable for direct integration into functional nanodevices. Crucially, the *n*T supramolecular structure confers unprecedented phase-dependent electronic tunability unattainable in amorphous or non-columnar analogues. The abrupt mobility enhancement during the Col_rec_–Col_hex_ phase transition directly links the carrier hopping pathways to tilted and parallel molecular packing. Moreover, extended π-conjugation synergistically enhances carrier transport, enabling solution-processable HTMs with dynamically tunable operating regimes. This synergistic combination of defect suppression, phase switchable behavior, and controlled carrier transport presents a highly viable and versatile approach for next-generation organic electronics.

## Author contributions

S. B. Chen: investigation, data curation, methodology, formal analysis, writing-original draft. X. Y. Du and Q. Q. Han: data curation, validation. J. J. Luo, F. Wang and J. M. Liu: supervision, validation. Y. Yu and X. H. Cheng: methodology, supervision, funding acquisition, writing–review & editing.

## Conflicts of interest

The authors declare no conflict of interest.

## Supplementary Material

SC-016-D5SC04039F-s001

## Data Availability

The data supporting this article have been included as part of the SI. See DOI: https://doi.org/10.1039/d5sc04039f.
