# Supplementary material for: Tilted vs. parallel assembly caused birefringent reversal in columnar phases of oligothiophene as well as applications for LEDs and hole transport materials
Source: Chem Sci. 2025 Aug 14;16(36):16851–66. doi: 10.1039/d5sc04039f (PMC12369646; doi:10.1039/d5sc04039f)
Supplement: SC-016-D5SC04039F-s001 [file SC-016-D5SC04039F-s001.pdf]

Supplementary Information (SI) for Chemical Science.
This journal is © The Royal Society of Chemistry 2025

supporting information

Tilted vs. parallel assembly caused birefringent reversal in columnar phases of oligothiophene as well as applications for LEDs and hole transport materials

Shibo Chen,[a] Xuyang Du,[a] Qingqing Han,[a] Jingjing Luo,[b] Fen Wang,[a] Jiaming Liu,[a] Yu Yang,[b] Xiaohong Cheng[a*]

[a] Key Laboratory of Medicinal Chemistry for Natural Resource, Ministry of Education; Yunnan Research & Development Center for Natural Products; School of Chemical Science and Technology, Yunnan University, Kunming, 650091, P.R. China. E-mail: xhcheng@ynu.edu.cn

[b] School of Materials and Energy, Yunnan University, Kunming 650091, P. R. China. E-mail: yuyang@ynu.edu.cn

Content

1. Additional Experimental Data S2
2. Material synthesis and analytical data S10
3. References S23

1. Additional Experimental Data

1.1 Experimental techniques

Polarizing Optical Microscopy (POM): Optical textures of all compounds were characterized by polarizing optical microscopy (Leica DM2700P) with the combination of a heating stage and controller (Linkam T9). Optical investigations were carried out under equilibrium conditions between two glass slides that were used without further treatment. A full wavelength retardation plate was used to determine the sign of birefringence.

DSC measurements: Transition enthalpies were obtained by differential scanning calorimetry (DSC), which was recorded on a DSC 200 F3 Maia calorimeter (NETZSCH) in sealed 30 μL aluminum pans with heating and cooling rates of 10 K/min under an N_2 stream; peak temperatures are given in Table 1.

X-ray diffraction: Identification of the phase type and determination of the lattice parameters were carried out by small and wide-angle X-ray diffraction (XRD, Rigaku Co., Tokyo, Japan) on a temperature-controlled heating stage. Analysis was conducted on a D/max-3B spectrometer with Cu Kα radiation.

Electron density reconstruction: Experimental diffractograms are fitted using Jade 6.0 to determine the positions and intensities of the diffraction peaks. The diffraction peaks are indexed based on their peak positions, and the lattice parameters and the space groups are subsequently determined. Once the diffraction intensities are measured and the corresponding plane group is determined, 2D electron density maps can be reconstructed based on the general formula:

$$E(xy) = \Sigma_{hk} \mathrm{sqrt}[I(hk)] \exp[i2\pi(hx+ky) +\phi_{hk}]$$

For the molecular structures considered in this work, the phase angle ϕ_{hk} can take up the values of 0 or π. The choice of a phase combination was initially made on the merit of each reconstructed electron density map obtained using the most intense reflections, combined with the additional knowledge of the molecules (molecular shape, length, volume of each part and the distribution of electron density among the different moieties).

Photophysical properties measurements: The UV-vis absorption and PL spectra were carried out on UV2600A UV-vis absorption spectrometer (UNICO, China) and Hitachi F-7000 fluorescence spectrometer (Hitachi, Japan).

The quantum yields of compounds in organic solvents were determined using quinolinium hydrogen sulphate in H_2SO_4 (Φ_{PL}=0.55) as standard and applying the following equation.[S1]

$$\Phi_{PL} = \Phi_{F_{st}} \frac{A_{st} F_x {n_x}^2}{A_x F_{st} {n_{st}}^2}$$

Φ_{PL}= the quantum yields of the samples; $\Phi_{F_{st}}$= the quantum yield of the standard; A = the absorbance of the solution; F = the integration of corrected fluorescence spectrum; n = the average refractive index of the solvent.

Variable-temperature PL measurements: The PL emission measurements under heating were

performed on thin films using a fluorescence spectrophotometer (Hitachi F-7000) equipped with a temperature-dependent accessory. Thin-film samples were prepared via spin-coating. Quartz substrates were first cleaned sequentially using a 5% soap solution, deionized water, acetone, and isopropyl alcohol in an ultrasonic cleaner for 20 minutes each. The cleaned substrates were then dried under vacuum. Next, the quartz glass surfaces were modified by UV-ozone treatment to remove any chemical residues remaining from the cleaning process. After 30 minutes of UV-ozone treatment, thin films of **4T** were prepared by spin-coating a solution of the compound in anhydrous chloroform (10 mg/mL) onto the quartz substrates at 1000 rpm for 60 s, forming an approximately 100 nm thick layer. The thickness of the layers was measured using a Dektak-XT stylus profiler. Subsequently, the sample films were annealed at 155 °C for 10 minutes to completely remove residual organic solvents. This annealing process also allowed the molecules to undergo gradual self-assembly, facilitating analysis of the spectral behavior of the LC phases at different temperatures. Finally, the prepared films were covered with another cleaned quartz substrate and mounted on the temperature-controlled accessory. The accessory containing the prepared sample was then placed into the fluorescence spectrophotometer. Ultimately, the sample was heated from room temperature at a rate of 10 °C/min, with PL spectra recorded at 20 °C intervals during heating until reaching the isotropic liquid state.

Theoretical calculation: Molecular models were built using Materials Studio 8.0 (Accelrys). Geometry optimization was performed using Gaussian 09 with B3LYP/6-311G (d) level.

Fabrication and characterization of device: To determine the carrier transport properties of the **1T-6T**, Hall devices were fabricated using the architecture: Quartz glass/**nT**/Ag. Quartz substrates were first cleaned sequentially using a 5% soap solution, deionized water, acetone, and isopropyl alcohol in an ultrasonic cleaner for 20 minutes each. The cleaned substrates were then dried under vacuum. Next, the quartz glass surfaces were modified by UV-ozone treatment to remove any chemical residues remaining from the cleaning process. After 30 minutes of UV-ozone treatment, thin films of **nT** were prepared by spin-coating a solution of the compounds in anhydrous chloroform (10 mg/mL) onto the quartz substrates at 1000 rpm for 60 s, forming an approximately 100 nm thick layer. The thickness of the layers was measured using a Dektak-XT stylus profiler. Subsequently, these films were annealed at specific temperatures for 10 minutes (90 °C for **1T**, 108 °C for **2T**, 113 °C for **3T,** 155 °C for **4T**, 158 °C for **5T**, 200 °C for **6T**). The devices were then transferred to a thermal evaporation system. 100 nm silver (Ag) top electrode were successively deposited on top of the active layer under a vacuum pressure of 2×10^{-6} mbar. The active device area was defined to be 0.09 cm^2 by a shadow mask. The electrical characteristic of the device was measured by Hall measurement system (East Changing, HT-100). Hall effect measurement was performed using a vanderborg test geometry.

Fabrication of thin films for PL measurements and POM surface analysis: Quartz substrates were first cleaned sequentially using a 5% soap solution, deionized water, acetone, and isopropyl alcohol in an ultrasonic cleaner for 20 minutes each. The cleaned substrates were then dried under vacuum. Next, the quartz glass surfaces were modified by UV-ozone treatment to remove any chemical residues remaining from the cleaning process. After 30 minutes of UV-ozone treatment, thin films of **nT** were prepared by spin-coating a solution of the compounds in anhydrous

chloroform (10 mg/mL) onto the quartz substrates at 1000 rpm for 60 s, forming an approximately 100 nm thick layer. The thickness of the layers was measured using a Dektak-XT stylus profiler. Subsequently, these films were annealed at specific temperatures for 10 minutes (90 °C for **1T**, 108 °C for **2T**, 113 °C for **3T,** 155 °C for **4T**, 158 °C for **5T**, 200 °C for **6T**). The specific annealing temperatures enable molecular self-assembly, thereby facilitating the formation of optimized thin films and the development of charge carrier transport pathways. The annealed film samples can be directly subjected to solid-state PL measurements and surface analysis using POM. Critically, films prepared in this manner are identical to those used in devices for measuring charge carrier transport properties, thus capable of reflecting the actual surface conditions in functional devices.

1.2 Additional experimental data

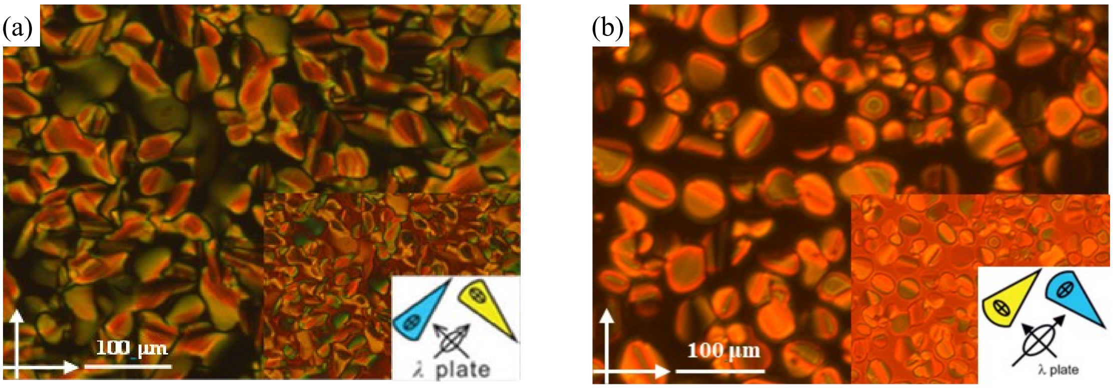

Fig. S1 (a) Polarizing optical microscopy textures of the Col_{hex}/*p*6*mm* phase of **3T** at 90 °C; (b) Polarizing optical microscopy textures of the Col_{rec}/*c*2*mm* phase of **6T** at 100 °C. Insets show texture with a λ-retarder plate.

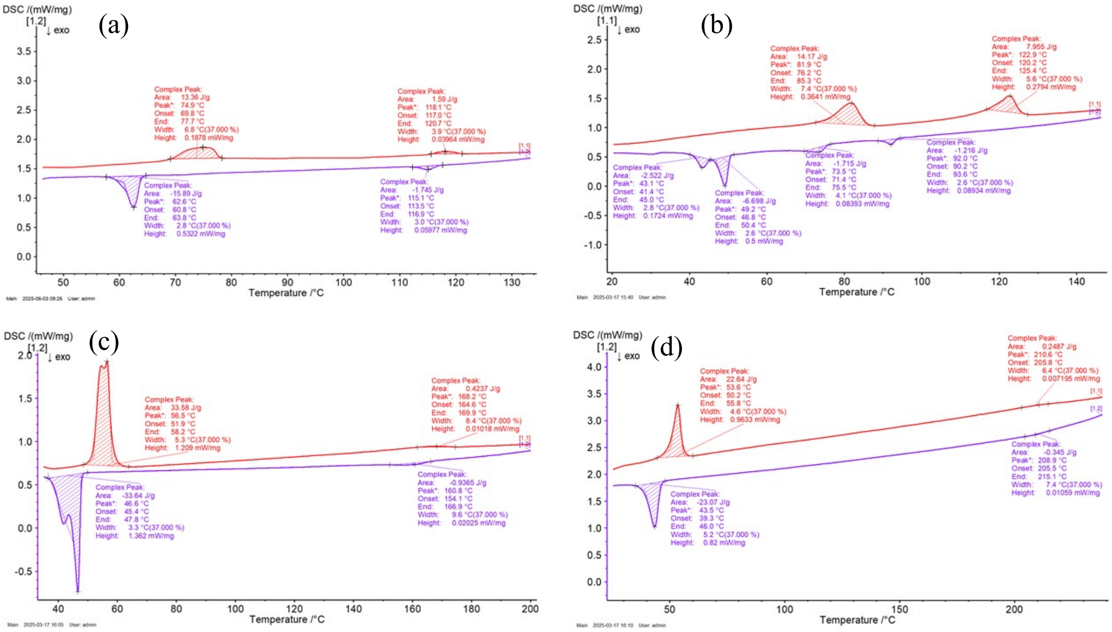

Fig. S2 DSC heating and cooling scans (10 K min^{-1}) of compounds **2T**(a), **3T**(b), **5T** (c) and **6T** (d). Red line represents heating process, purple line represents cooling process.

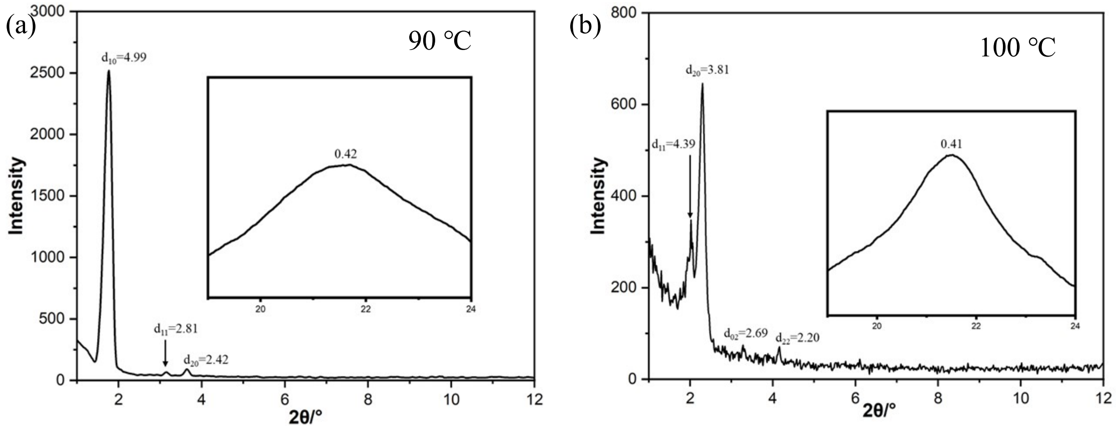

Fig. S3 XRD patterns of the $Col_{rec}/p6mm$ phase of **3T** (a) and $Col_{rec}/c2mm$ phase of **6T** (b).

Table S1 Experimental and calculated *d*-spacings and relative integrated intensities, for the $Col_{hex}/p6mm$ phase of **2T** at 110 °C. All intensities values are Lorentz corrected with correction for multiplicity.

(*hk*)	$2\theta_{obs.}$ (°)	$d_{obs.}$ -spacing(nm)	$d_{cal.}$-spacing(nm)	*Intensity*
10	1.91	4.62	4.59	100
11	3.31	2.67	2.65	2.7
20	3.88	2.27	2.29	2.9
a_{hex} =5.30 nm				

[a](*hk*): assigned indices for 2D phases.

Table S2 Experimental and calculated *d*-spacings and relative integrated intensities, for the $Col_{hex}/p6mm$ phase of **3T** at 90 °C. All intensities values are Lorentz corrected with correction for multiplicity.

(*hk*)	$2\theta_{obs.}$ (°)	$d_{obs.}$ -spacing(nm)	$d_{cal.}$-spacing(nm)	*Intensity*
10	1.77	4.99	4.84	100
11	3.14	2.81	2.80	2.3
20	3.64	2.42	2.42	3.4
a_{hex} =5.59 nm				

Table S3 Experimental and calculated *d*-spacings and relative integrated intensities, for the $Col_{hex}/p6mm$ phase of **4T** at 80 °C. All intensities values are Lorentz corrected with correction for multiplicity.

(*hk*)	$2\theta_{obs.}$ (°)	$d_{obs.}$ -spacing(nm)	$d_{cal.}$-spacing(nm)	*Intensity*
10	1.69	5.22	5.20	100
11	2.95	2.99	3.00	15.6
20	3.39	2.60	2.60	16.4
a_{hex} =6.00 nm				

Table S4 Experimental and calculated *d*-spacings and relative integrated intensities, for the $Col_{rec}/c2mm$ phase of **4T** at 150 °C. All intensities values are Lorentz corrected with correction for multiplicity.

(hk)	$2\theta_{obs.}$ (°)	$d_{obs.}$ -spacing(nm)	$d_{cal.}$-spacing(nm)	*Intensity*
11	2.24	3.94	3.94	80.7
20	2.48	3.56	3.56	100
02	3.73	2.36	2.36	13.4
22	4.48	1.97	1.97	14.8
a_{rec} =7.12 nm, b_{rec} =4.72 nm				

Table S5 Experimental and calculated *d*-spacings and relative integrated intensities, for the $Col_{rec}/c2mm$ phase of **5T** at 100 °C. All intensities values are Lorentz corrected with correction for multiplicity.

(hk)	$2\theta_{obs.}$ (°)	$d_{obs.}$ -spacing(nm)	$d_{cal.}$-spacing(nm)	*Intensity*
11	2.13	4.15	4.15	56.0
20	2.49	3.55	3.55	100
02	3.45	2.56	2.56	12.6
22	4.25	2.08	2.08	10.8
a_{rec} =7.10 nm, b_{rec} =5.12 nm				

Table S6 Experimental and calculated *d*-spacings and relative integrated intensities, for the $Col_{rec}/c2mm$ phase of **6T** at 100 °C. All intensities values are Lorentz corrected with correction for multiplicity.

(hk)	$2\theta_{obs.}$ (°)	$d_{obs.}$ -spacing(nm)	$d_{cal.}$-spacing(nm)	*Intensity*
11	2.01	4.39	4.39	53.9
20	2.32	3.81	3.81	100
02	3.39	2.69	2.69	11.6
22	4.02	2.20	2.20	11.0
a_{rec} =7.62 nm, b_{rec} =5.37 nm				

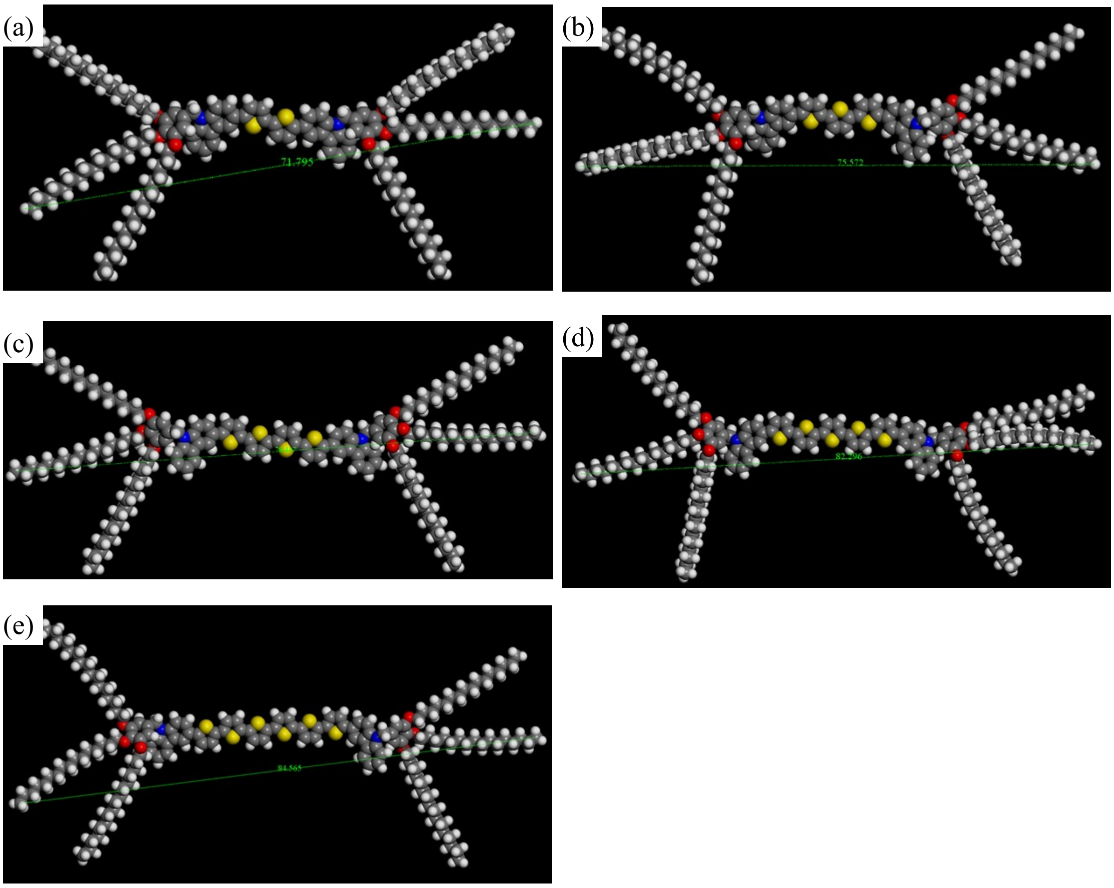

Fig. S4 The maximum extended molecular length of **2T**, **3T**, **4T**, **5T** and **6T** were measured by Materials Studio.

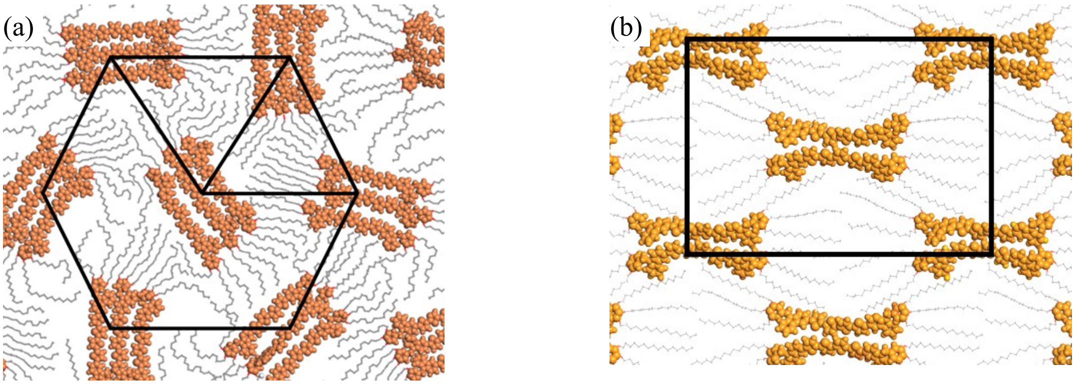

Fig. S5 (a) Molecular dynamics model of $Col_{hex}/p6mm$ phase; (b)Molecular dynamics model of $Col_{rec}/c2mm$ phase.

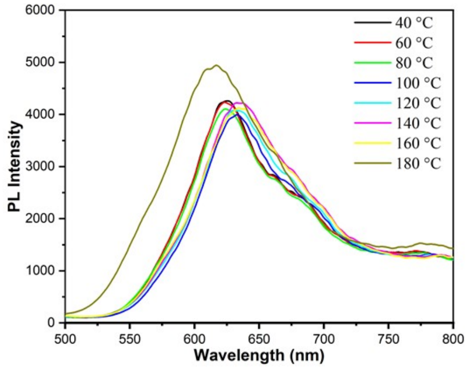

Fig. S6 PL spectra of compound **4T** thin film during heating processes.

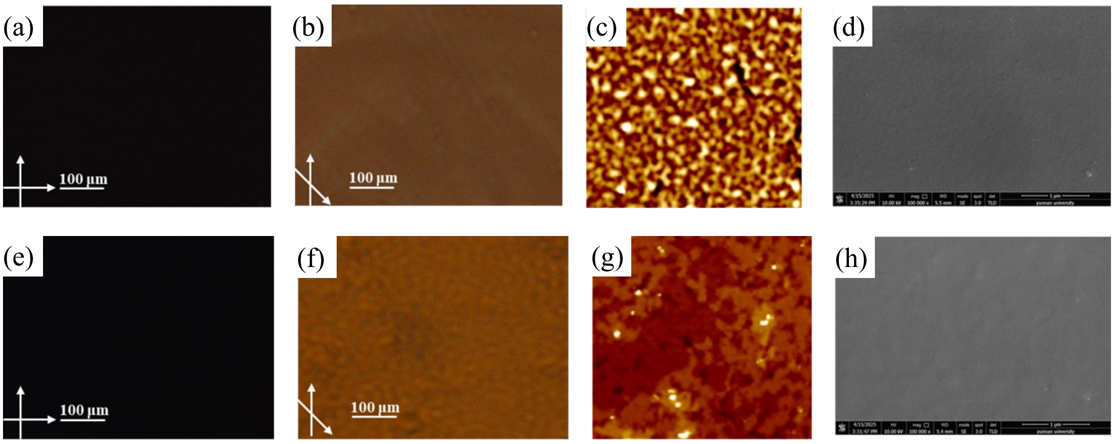

Fig. S7 (a and e) POM images (100 nm thin film) of **5T** and **6T** at room temperature; arrows represent the relative direction of the polarizer and analyzer (polarizer and analyzer 90° cross to each other); (b and f) POM image of the same slide (45°); (c and g) AFM images of the **5T** and **6T** thin film; (d and h) SEM images elegantly show cases the continuity and conformity of **5T** and **6T**.

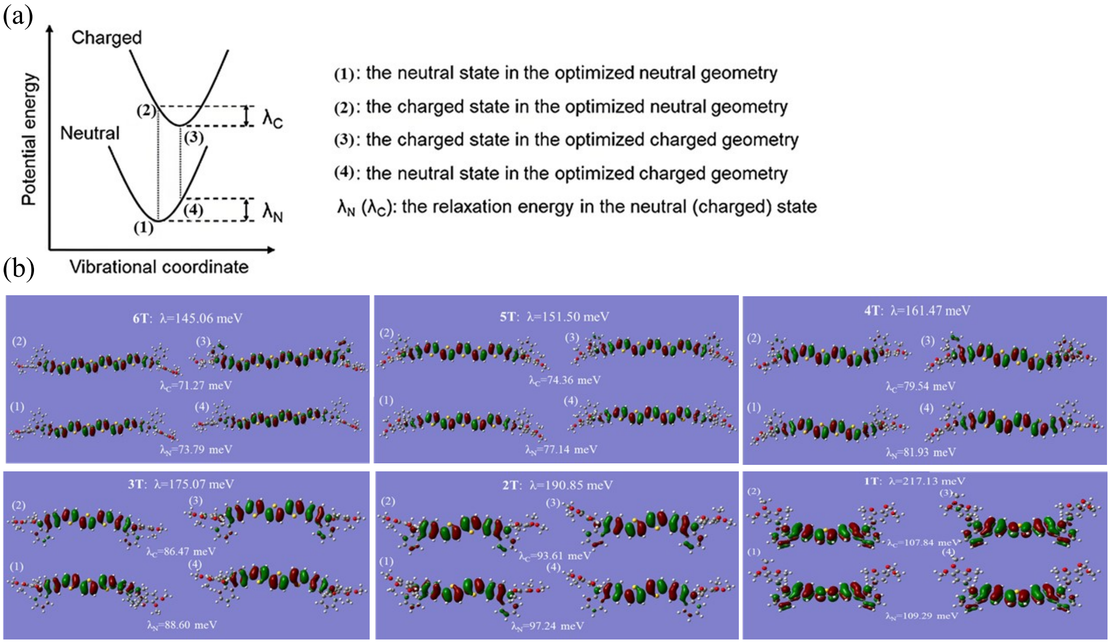

Fig. S8 (a) A schematic of adiabatic potential energy surfaces corresponding to the ionization process. (b) Reorganization energies of the **nT**, with the HOMOs in each state (calculated at B3LYP/6 311G level of theory).

Table S7 Reorganization energy data of **nT**.

**Comp.**	λ_N (emV)	λ_C (emV)	λ (emV)^a
**1T**	109.29	107.84	217.13
**2T**	97.24	93.61	190.85
**3T**	88.60	86.47	175.07
**4T**	81.93	79.54	161.47
**5T**	77.14	74.36	151.50
**6T**	73.79	71.27	145.06

$\lambda=\lambda_N + \lambda_C$

1.3 Additional discussion

The structure of the spin-coated films of **4T-6T** at room temperature (25 °C) were analyzed using XRD patterns (Fig. S9). The results demonstrate that the small-angle region ($2\theta \approx 1°$-$10°$) exhibits characteristic reflection ratios identical to those of their LC phases (namely, hexagonal columnar stacking for **4T** at low temperature and rectangular columnar stacking for **5T** and **6T**). This confirms the retention of long-range columnar stacking order within the films, while in the wide-angle region ($2\theta > 10°$), the diffuse halo characteristic of the LC phase (molecular positional disorder and high dynamic behavior) disappears, and distinct crystalline peaks emerge, indicating the occurrence of crystallization in the compounds. These structural characteristics are crucial for the carrier transport mechanism, primarily governed by the dominant columnar framework. Additionally, the characteristic reflection ratios observed in small-angle XRD confirm that the annealed films retain the quasi-one-dimensional carrier transport pathways typical of columnar LC phases, forming the structural basis for high mobility. While the crystalline peaks in the wide-angle region reflect changed intracolumnar molecular packing order, they do not disrupt the overall columnar structure. Such "ordered columnar stacking" is critically important in organic semiconductors. In such systems, carrier transport properties typically remain within the same order of magnitude as those that are observed in the pure LC phase. This stability arises because carrier transport occurs predominantly along the columnar axis, rendering it relatively insensitive to enhance local intracolumnar order.[S2] Consequently, the carrier transport properties of **4T-6T** measured at room temperature are essentially identical to those observed in the LC temperature range (Fig. 17).

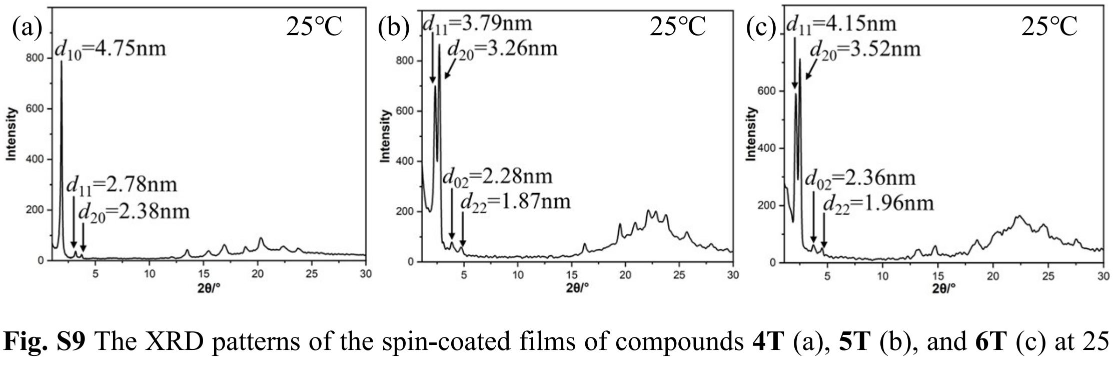

Fig. S9 The XRD patterns of the spin-coated films of compounds **4T** (a), **5T** (b), and **6T** (c) at 25

°C.

2. Material synthesis and analytical data

2.1 General remarks

The structures of the compounds are shown in Scheme S1. Reactions requiring an inert gas atmosphere were conducted under nitrogen and the glassware was oven-dried (120 °C). Tetrahydrofuran (THF) was distilled from sodium prior to use. Commercially available chemicals were used as received. ^{1}H NMR and ^{13}C NMR spectra were recorded on a Bruker-DRX-400 spectrometer. Thin-layer chromatography was performed on aluminum plates precoated with 5735 silica gel 60 PF254 (Merck). Column chromatography was performed on Merck silica gel 60 (300-400 mesh). Mass spectra were recorded with a Bruker rapifleX MALDI-TOF/TOF. The melting points (m.p.) of solid intermediates were determined by melting point meter (Jiahang JH30, China).

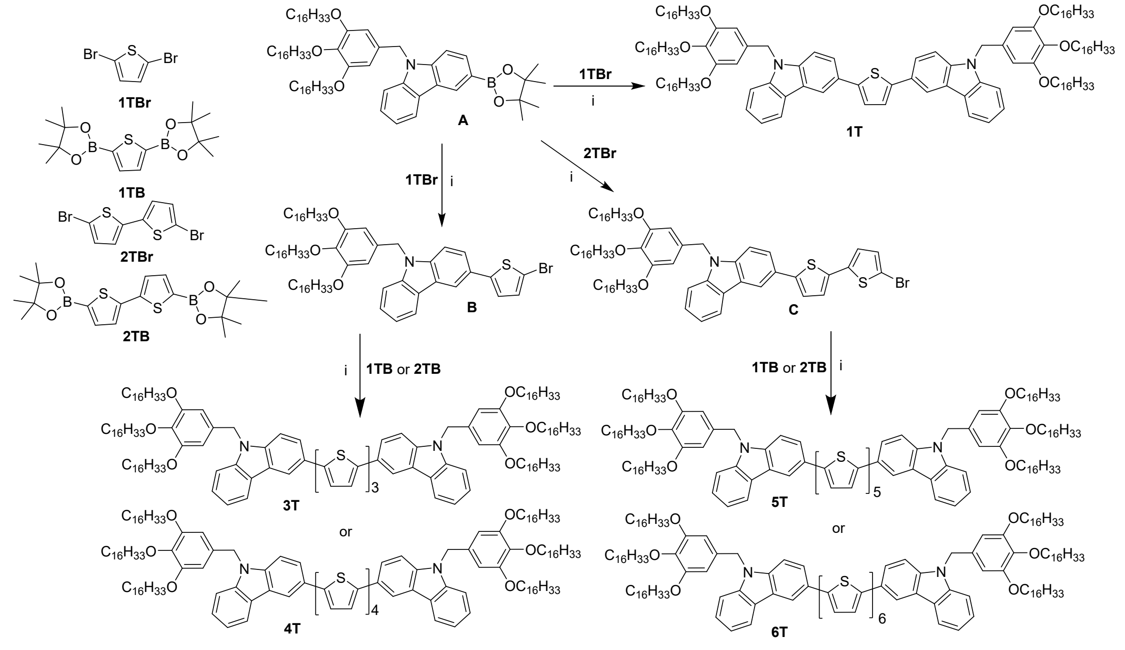

Scheme S1 Synthesis of **nT**. Reagents and conditions: $Pd(PPh_3)_4$, K_2CO_3, H_2O, THF, N_2, 80 °C, 12 h.

Compound **A**[S3] was synthesized according to the references and the corresponding NMR data is consistent with those reported in reference.

Synthesis of 1T and 2T

A mixture of **A** (200 mg, 0.18 mmol), **1TBr** (22 mg, 0.09 mmol) or **2TBr** (29 mg, 0.09 mmol), $Pd(PPh_3)_4$ (catalytic amount), THF (25 mL) and K_2CO_3 solution (1.8 mmol in 10 mL water) was stirred under N_2 atmosphere at reflux for 12 h. After cooling to room temperature, the solvent was removed and the residue was extracted with CH_2Cl_2 (3 × 25 mL). The combined organic layers were washed with water (3 × 25 mL). After drying over anhydrous $MgSO_4$, filtration and evaporation of the solvent. The residue was purified by column chromatography (eluent: petroleum ether: ethyl acetate = 15: 1).

1T, Yield: 156 mg, 85 %; white solid, m.p.97-99 °C: ^{1}H NMR ($CDCl_3$, 400 MHz), δ (ppm): 8.39 (s, 2H, 2Ar-**H**), 8.19-8.17 (d, *J*=7.6 Hz, 2H, 2Ar-**H**), 7.77-7.75 (d, *J*=7.6 Hz, 2H, 2Ar-**H**), 7.47-7.44 (t, *J*=7.2 Hz, 2H, 2Ar-**H**), 7.40-7.36 (m, 6H, 6Ar-**H**), 7.30-7.28 (d, *J*=7.6 Hz, 2H, 2Ar-**H**), 6.35 (s, 4H, 4Ar-**H**), 5.42 (s, 4H, 2ArC**H**$_2$N), 3.90-3.78 (m,12H, 6ArOC**H**$_2$), 1.72-1.63 (m, 12H, 6ArOCH$_2$C**H**$_2$), 1.43-1.36 (m, 12H, 6ArOCH$_2$CH$_2$C**H**$_2$), 1.24 (m, 144H, 72C**H**$_2$), 0.88-0.85 (t, 18H, 3C**H**$_3$). ^{13}C NMR ($CDCl_3$; 100 MHz): 153.5, 143.8, 141.3, 140.3, 137.6, 132.1, 126.3, 126.2, 124.1, 123.5, 123.0, 122.9, 120.6, 119.5, 117.4, 109.4, 109.3, 104.9, 73.4, 69.1, 47.1, 32.0, 30.3, 29.8, 29.8, 29.7, 29.7, 29.6, 29.5, 29.4, 29.3, 26.1, 26.1, 22.7, 14.2. HRMS spectrum of **1T**, MALDI-TOF-MS m/z: Found 2036.6863 $[M+H]^+$. Calculated For $C_{138}H_{222}N_2O_6S$: 2035.6849.

2T, Yield: 152 mg, 80 %; green solid: ^{1}H NMR ($CDCl_3$, 400 MHz), δ (ppm): 8.35 (s, 2H, 2Ar-**H**), 8.18-8.16 (d, 2H, *J*=8.0 Hz, 2Ar-**H**), 7.72-7.70 (d, 2H, *J*=8.0 Hz, 2Ar-**H**), 7.48-7.44 (t, 2H, *J*=7.2 Hz, 2Ar-**H**), 7.39-7.38 (d, 2H, *J*=4.0 Hz, 2Ar-**H**), 7.37-7.36 (d, 2H, *J*=4.0 Hz, 2Ar-**H**), 7.31-7.26 (m, 4H, 4Ar-**H**), 7.22-7.21 (d, 2H, *J*=4.0 Hz, 2Ar-**H**), 6.35 (s, 4H, 4Ar-**H**), 5.41 (s, 4H, 2ArC**H**$_2$N), 3.91-3.78 (m, 12H, 6ArOC**H**$_2$), 1.73-1.64 (m, 12H, 6ArOCH$_2$C**H**$_2$), 1.44-1.36 (m, 12H, 6ArOCH$_2$CH$_2$C**H**$_2$), 1.26 (m, 144H, 72C**H**$_2$), 0.90-0.86 (t, 18H, 3C**H**$_3$). ^{13}C NMR ($CDCl_3$; 100 MHz): 153.5, 144.2, 141.3, 140.4, 137.6, 135.9, 132.0, 126.3, 125.8, 124.2, 124.1, 123.6, 123.0, 122.7, 120.6, 119.6, 117.5, 109.4, 109.3, 104.9, 73.4, 69.1, 47.1, 32.0, 30.3, 29.8, 29.7, 29.7, 29.5, 29.4, 29.3, 26.1, 22.7, 14.2. HRMS spectrum of **2T**. MALDI-TOF-MS m/z: Found 2118.6846 $[M+H]^+$. Calculated For $C_{142}H_{224}N_2O_6S_2$: 2117.6726.

Synthesis of B and C

A mixture of **A** (800 mg, 0.72 mmol), **1TBr** (172 mg, 0.72 mmol) or **2TBr** (232 mg, 0.72 mmol), $Pd(PPh_3)_4$ (catalytic amount), THF (25 mL) and K_2CO_3 solution (7.2 mmol in 10 mL water) was stirred under N_2 atmosphere at reflux for 12 h. After cooling to room temperature, the solvent was removed and the residue was extracted with CH_2Cl_2 (3 × 25 mL). The combined organic layers were washed with water (3 × 25 mL). After drying over anhydrous $MgSO_4$, filtration and evaporation of the solvent. The residue was purified by column chromatography (eluent: petroleum ether: ethyl acetate = 10: 1).

B, Yield: 476 mg, 58 %; white solid, m.p. 92-94 °C: ^{1}H NMR (400 MHz, $CDCl_3$) δ (ppm) 8.24 (s, 1H, 1Ar-**H**), 8.15-8.13 (d, *J*=7.6 Hz, 1H, 1Ar-**H**), 7.60-7.57 (d, *J*=10.4 Hz, 1H, 1Ar-**H**), 7.45-7.43 (d, *J*=7.6 Hz, 1H, 1Ar-**H**), 7.39 (s, 2H, 2Ar-**H**), 7.36-7.27 (m, 1H, 1Ar-**H**), 7.08-7.04 (m, 2H, 2Ar-**H**), 6.32 (s, 2H, 2Ar-**H**), 5.40 (s, 2H, ArC**H**$_2$N), 3.89-3.76 (m, 6H, 3ArOC**H**$_2$), 1.74-1.62 (m, 6H, 3ArOCH$_2$C**H**$_2$), 1.42-1.40 (m, 6H, 3ArOCH$_2$CH$_2$C**H**$_2$), 1.24 (m, 72H, 36C**H**$_2$), 0.89-0.86 (t, 9H, 3C**H**$_3$).

C, Yield: 466 mg, 53 %; white solid, m.p. 124-126 °C: ^{1}H NMR (400 MHz, $CDCl_3$) δ (ppm) 8.15 (s, 1H, 1Ar-**H**), 7.78-7.66 (m, 1H, 1Ar-**H**), 7.46-7.44 (d, *J*=7.2 Hz, 1H, 1Ar-**H**), 7.39-7.37 (d, *J*=8.0 Hz, 1H, 1Ar-**H**), 7.35 (s, 2H, 2Ar-**H**), 7.30 (s, 1H, 1Ar-**H**), 7.28-7.23 (m, 1H, 1Ar-**H**),7.11-7.10 (d, *J*=4.0 Hz, 1H, 1Ar-**H**), 6.99-6.94 (m, 2H, 2Ar-**H**), 6.33 (s, 2H, 2Ar-**H**), 5.41 (s, 2H, ArC**H**$_2$N), 3.90-3.77 (m, 6H, 3ArOC**H**$_2$), 1.72-1.64 (m, 6H, 3ArOCH$_2$C**H**$_2$), 1.43-1.34 (m, 6H, 3ArOCH$_2$CH$_2$C**H**$_2$), 1.24 (m, 72H, 36C**H**$_2$), 0.89-0.86 (t, 9H, 3C**H**$_3$).

Synthesis of 3T and 4T

A mixture of **B** (200 mg, 0.18 mmol), **1TB** (30 mg, 0.09 mmol) or **2TB** (38 mg, 0.09 mmol), $Pd(PPh_3)_4$ (catalytic amount), THF (25 mL) and K_2CO_3 solution (1.8 mmol in 10 mL water) was stirred under N_2 atmosphere at reflux for 12 h. After cooling to room temperature, the solvent was removed and the residue was extracted with CH_2Cl_2 (3 × 25 mL). The combined organic layers were washed with water (3 × 25 mL). After drying over anhydrous $MgSO_4$, filtration and evaporation of the solvent. The residue was purified by column chromatography (eluent: petroleum ether: ethyl acetate = 8: 1).

3T, Yield: 162 mg, 82 %; yellow solid: ^{1}H NMR (400 MHz, $CDCl_3$) δ (ppm): 8.34 (s, 2H, 2Ar-**H**), 8.17-8.16 (d, *J*=4.8 Hz, 2H, 2Ar-**H**), 7.70-7.69 (d, *J*=4.8 Hz, 2H, 2Ar-**H**), 7.47-7.45 (t, *J*=4.8 Hz, 2H, 2Ar-**H**), 7.39-7.36 (m, 4H, 4Ar-**H**), 7.29-7.27 (d, *J*=9.6 Hz, 4H, 4Ar-**H**), 7.19 (s, 2H, 2Ar-**H**), 7.19 (s, 2H, 2Ar-**H**), 6.34 (s, 4H, 4Ar-**H**), 5.41 (s, 4H, 2ArC**H**$_2$N), 3.90-3.78 (m,12H, 6ArOC**H**$_2$), 1.72-1.64 (m, 12H, 6ArOCH$_2$C**H**$_{2)}$, 1.43-1.28 (m, 12H, 6ArOCH$_2$CH$_2$C**H**$_2$), 1.25 (m, 144H, 72C**H**$_2$), 0.89-0.87 (t, 18H, 3C**H**$_3$). ^{13}C NMR ($CDCl_3$; 100 MHz): 153.5, 143.8, 141.3, 140.3, 137.6, 132.1, 126.3, 126.2, 124.1, 123.5, 123.0, 123.0, 120.6, 119.5, 117.4, 109.4, 109.3, 104.9, 73.4, 69.1, 47.1, 32.0, 30.3, 29.8, 29.8, 29.7, 29.7, 29.6, 29.5, 29.4, 29.3, 26.1, 16.1, 22.7, 14.2. HRMS spectrum of **3T**. MALDI-TOF-MS m/z: Found 2200.65763 $[M+H]^+$. Calculated For $C_{146}H_{226}N_2O_6S_3$: 2199.6603.

4T, Yield: 164 mg, 80 %; orange solid: ^{1}H NMR (400 MHz, $CDCl_3$) δ (ppm) 8.33 (s, 2H, 2Ar-**H**), 8.17-8.15 (d, *J*=7.6 Hz, 2H, 2Ar-**H**), 7.70-7.68 (d, *J*=8.4 Hz, 2H, 2Ar-**H**), 7.48-7.44 (t, *J*=7.6 Hz, 2H, 2Ar-**H**), 7.39-7.35 (m, 4H, 4Ar-**H**), 7.30-7.26 (m, 4H, 4Ar-**H**), 7.18 (s, 2H, 2Ar-**H**), 7.12 (s, 4H, 4Ar-**H**), 6.33 (s, 4H, 4Ar-**H**), 5.40 (s, 4H, 2ArC**H**$_2$N), 3.90-3.77 (m,12H, 6ArOC**H**$_2$), 1.74-1.63 (m, 12H, 6ArOCH$_2$C**H**$_{2)}$, 1.43-1.35 (m, 12H, 6ArOCH$_2$CH$_2$C**H**$_2$), 1.25 (m, 144H, 72C**H**$_2$), 0.90-0.86 (t, 18H, 3C**H**$_3$). ^{13}C NMR ($CDCl_3$; 100 MHz): 153.4, 144.8, 141.3, 140.5, 137.6, 136.6, 135.6, 135.2, 132.0, 126.3, 125.6, 124.7, 124.2, 124.1, 123.9, 123.6, 122.9, 122.7, 120.6, 119.6, 117.5, 109.5, 109.3, 104.9, 73.4, 69.1, 47.0, 32.0, 31.6, 30.3, 29.8, 29.7, 29.7, 29.6, 29.4, 29.4, 29.3, 26.1, 26.1, 22.7, 14.2. HRMS spectrum of **4T**. MALDI-TOF-MS m/z: Found 2282.6555 $[M+H]^+$. Calculated For $C_{150}H_{228}N_2O_6S_4$: 2281.6480.

Synthesis of 5T and 6T

A mixture of **C** (200 mg, 0.18 mmol), **1TB** (30 mg, 0.09 mmol) or **2TB** (38 mg, 0.09 mmol), $Pd(PPh_3)_4$ (catalytic amount), THF (25 mL) and K_2CO_3 solution (1.8 mmol in 10 mL water) was stirred under N_2 atmosphere at reflux for 12 h. After cooling to room temperature, the solvent was removed and the residue was extracted with CH_2Cl_2 (3 × 25 mL). The combined organic layers were washed with water (3 × 25 mL). After drying over anhydrous $MgSO_4$, filtration and evaporation of the solvent. The residue was purified by column chromatography (eluent: petroleum ether: ethyl acetate = 7: 1).

5T, Yield: 170 mg, 80 %; red solid: ^{1}H NMR (400 MHz, $CDCl_3$) δ (ppm) 8.32 (s, 2H, 2Ar-**H**), 8.17-8.15 (d, *J*=7.6 Hz, 2H, 2Ar-**H**), 7.70-7.68 (m, 2H, 2Ar-**H**), 7.48-7.44 (t, *J*=7.6 Hz, 2H, 2Ar-**H**), 7.39-7.36 (m, 4H, 4Ar-**H**), 7.30-7.26 (m, 4H, 4Ar-**H**), 7.19 (s, 2H, 2Ar-**H**), 7.18-7.10 (m, 6H, 6Ar-**H**), 6.33 (s, 4H, 4Ar-**H**), 5.41 (s, 4H, 2ArC**H**$_2$N), 3.90-3.77 (m, 12H, 6ArOC**H**$_2$), 1.72-1.63 (m, 12H,

6ArOCH$_2$C**H**$_2$), 1.43-1.29 (m, 12H, 6ArOCH$_2$CH$_2$C**H**$_2$), 1.24 (m, 144H, 72C**H**$_2$), 0.89-0.86 (t, 18H, 3C**H**$_3$). ^{13}C NMR (CDCl$_3$; 100 MHz): 153.4, 141.3, 140.5, 137.6, 136.7, 135.4, 135.1, 132.6, 126.1, 125.6, 124.7, 124.1, 124.2, 124.1, 123.9, 123.6, 122.9, 122.7, 120.6, 119.6, 117.5, 109.4, 109.3, 104.9, 73.4, 69.1, 47.0, 32.0, 30.3, 29.8, 29.7, 29.7, 29.6, 29.5, 29.4, 29.3, 26.1, 26.1, 22.7, 14.2. HRMS spectrum of **5T**, MALDI-TOF-MS m/z: Found 2364.6312 [M+H]$^+$. Calculated For $C_{154}H_{230}N_2O_6S_5$: 2363.6357.

6T, Yield: 165 mg, 75 %; red solid: ^{1}H NMR (400 MHz, CDCl$_3$) δ (ppm) 8.33 (s, 2H, 2Ar-**H**), 8.17-8.15 (d, *J*=7.6 Hz, 2H, 2Ar-**H**), 7.70-7.68 (d, *J*=8.0 Hz, 2H, 2Ar-**H**), 7.47-7.44 (t, *J*=7.2 Hz, 2H, 2Ar-**H**), 7.39-7.36 (m, 4H, 4Ar-**H**), 7.30-7.26 (m, 5H, 5Ar-**H**), 7.19 (s, 2H, 2Ar-**H**), 7.11-7.10 (s, 7H, 7Ar-**H**), 6.33 (s, 4H, 4Ar-**H**), 5.41 (s, 4H, 2ArC**H**$_2$N), 3.90-3.77 (m, 12H, 6ArOC**H**$_2$), 1.70-1.64 (m, 12H, 6ArOCH$_2$C**H**$_2$), 1.43 (m, 12H, 6ArOCH$_2$CH$_2$C**H**$_2$), 1.24 (m, 144H, 72C**H**$_2$), 0.89-0.86 (t, 18H, 3C**H**$_3$). ^{13}C NMR (CDCl$_3$; 100 MHz): 153.4, 144.8, 141.3, 140.5, 137.6, 136.6, 135.6, 135.2, 132.0, 126.3, 125.6, 124.7, 124.3, 124.1, 123.9, 123.6, 122.9, 122.7, 120.6, 119.6, 117.6, 109.5, 109.3, 104.9, 73.4, 69.1, 47.1, 32.0, 30.3, 29.8, 29.7, 29.7, 29.6, 29.4, 29.4, 29.3, 26.1, 26.0, 22.7, 14.2. HRMS spectrum of **6T**, MALDI-TOF-MS m/z: Found 2446.6243 [M+H]$^+$. Calculated For $C_{158}H_{232}N_2O_6S_6$: 2445.6235.

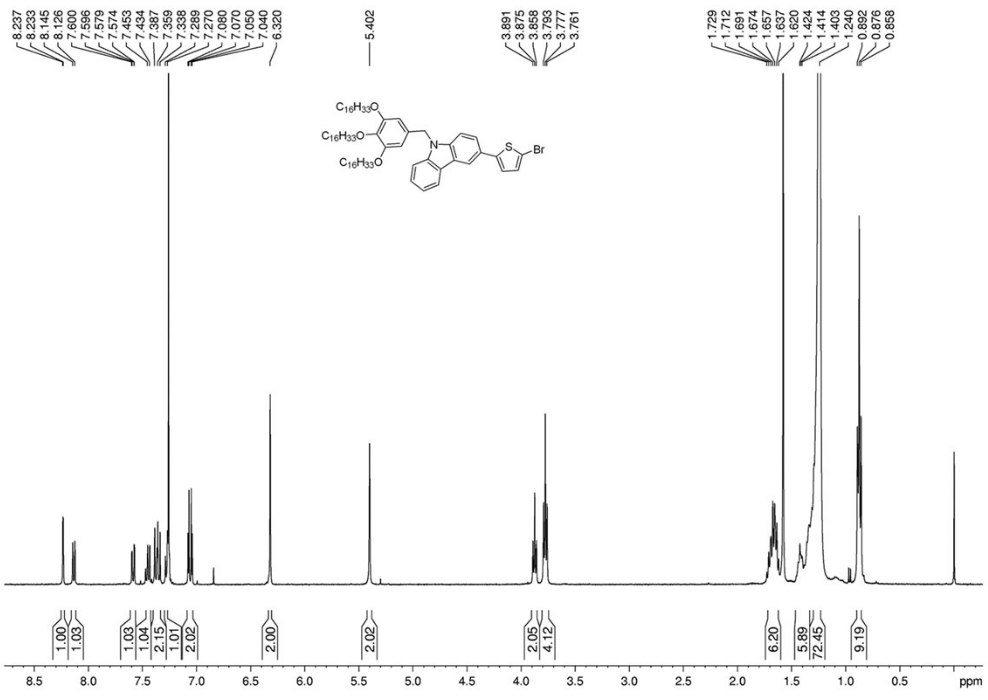

Fig. S10 ^{1}H NMR (CDCl$_3$, 400 MHz ppm) spectrum of **B**.

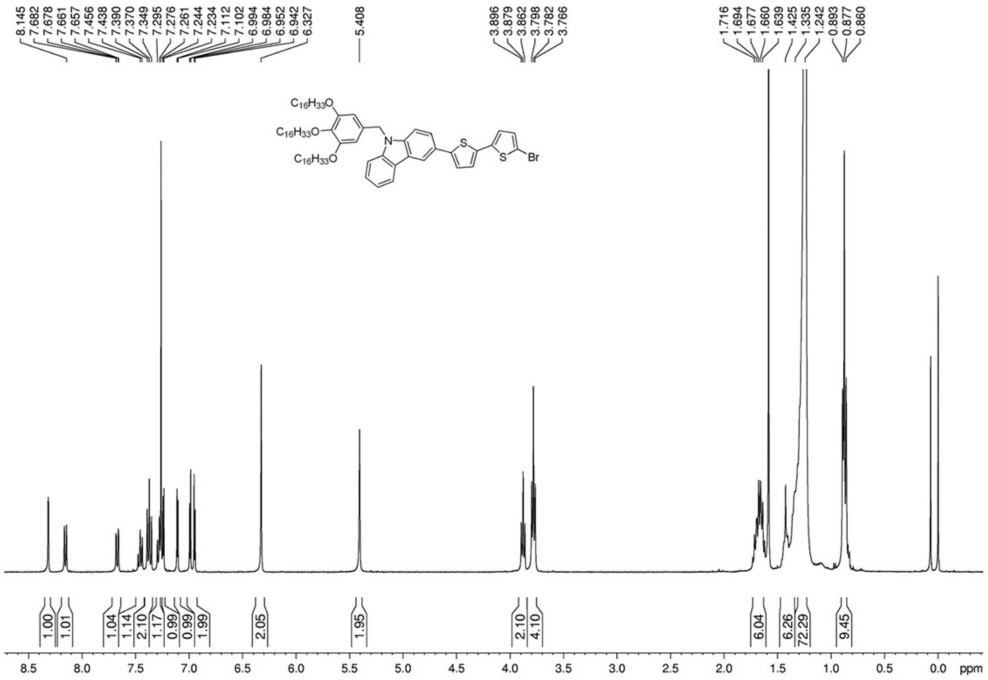

Fig. S11 ^{1}H NMR ($CDCl_3$, 400 MHz ppm) spectrum of representative compound **C**.

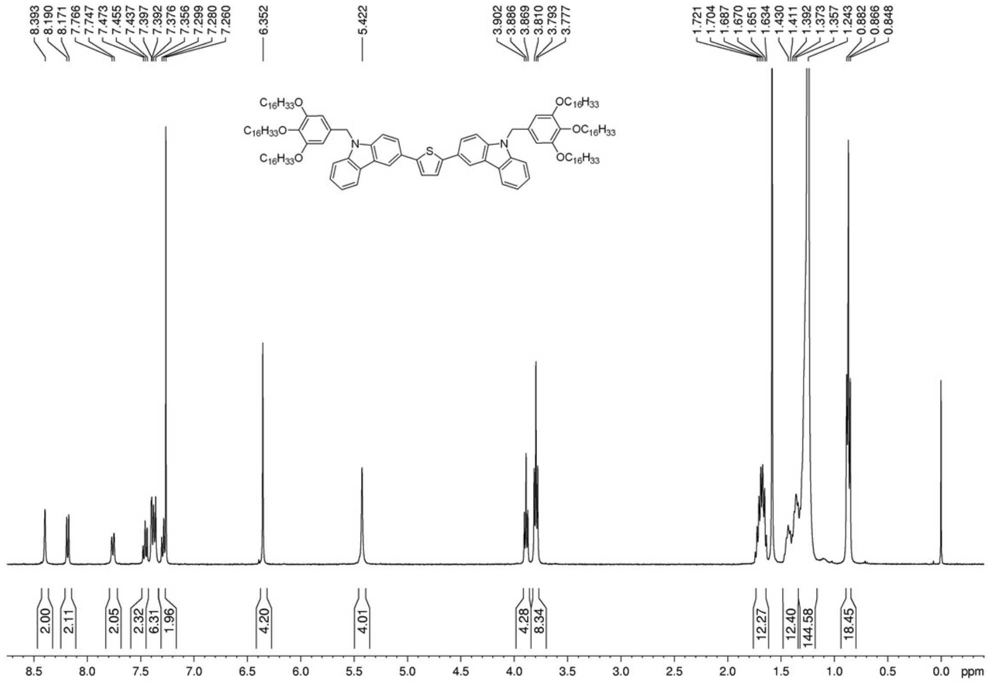

Fig. S12 ^{1}H NMR ($CDCl_3$, 400 MHz ppm) spectrum of **1T**.

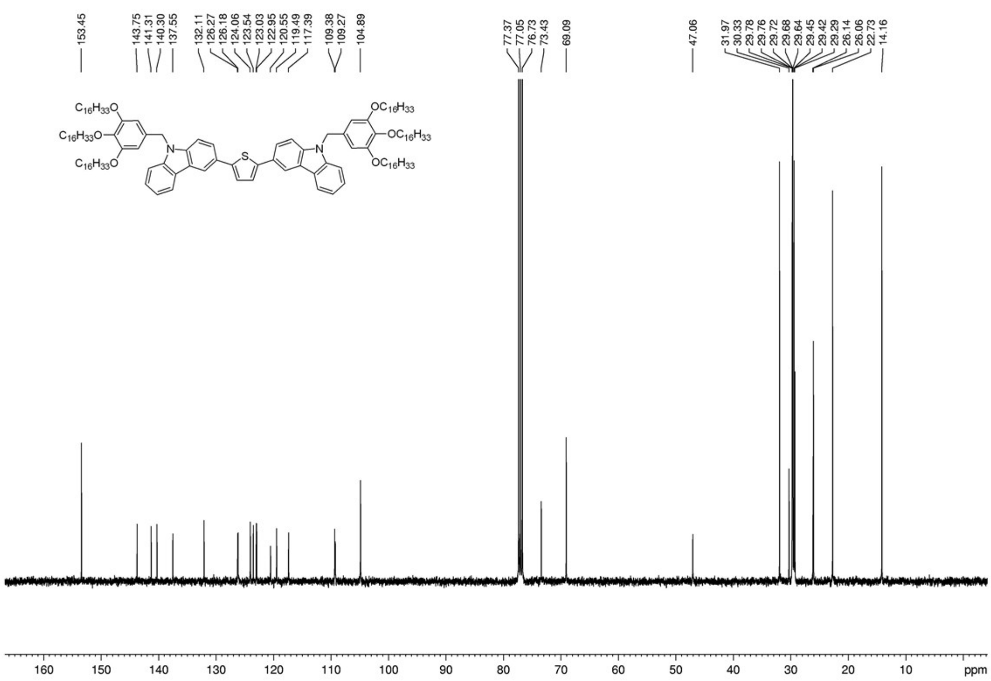

Fig. S13 ^{13}C NMR ($CDCl_3$, 100 MHz ppm) spectrum of **1T.**

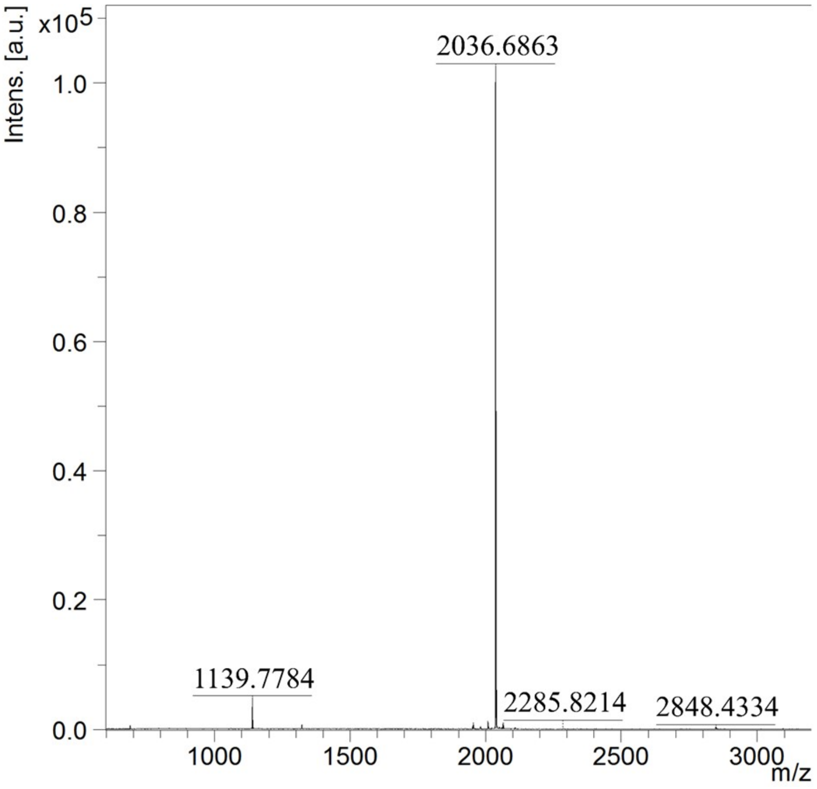

Fig. S14 HRMS spectrum of **1T**. MALDI-TOF-MS m/z: Found 2036.6863 $[M+H]^+$. Calculated For $C_{138}H_{222}N_2O_6S$: 2035.6849.

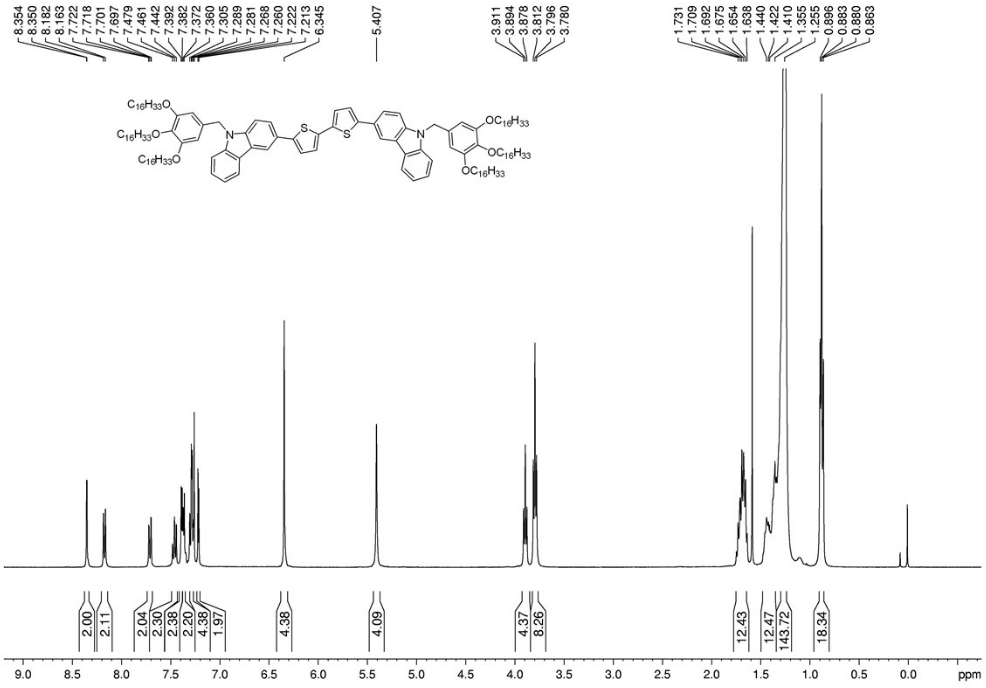

Fig. S15 ^{1}H NMR ($CDCl_3$, 400 MHz ppm) spectrum of **2T**.

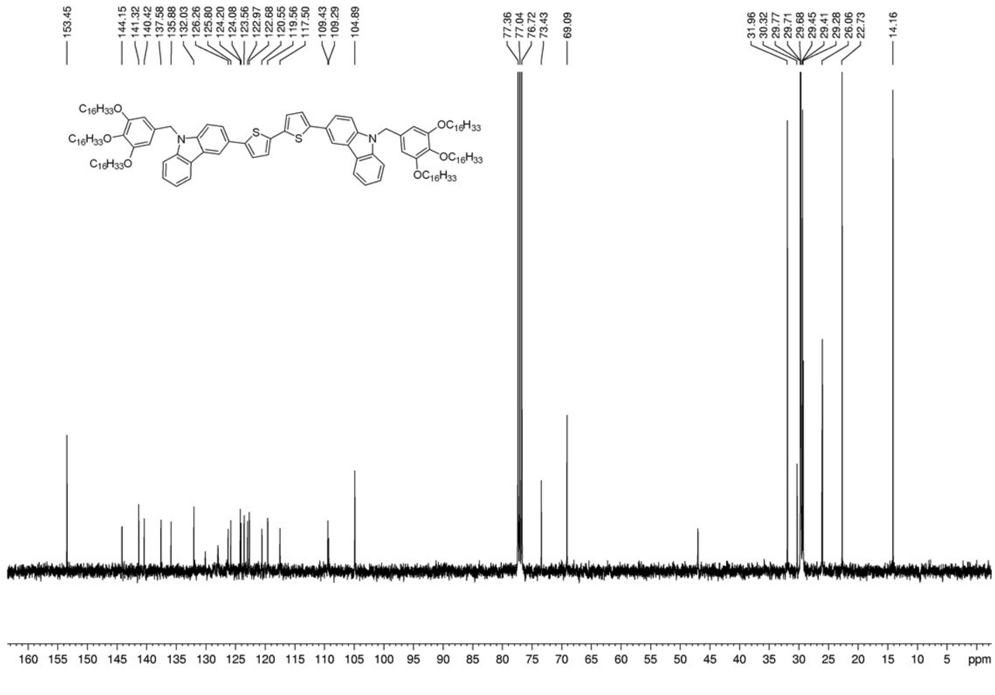

Fig. S16 ^{13}C NMR ($CDCl_3$, 100 MHz ppm) spectrum of **2T.**

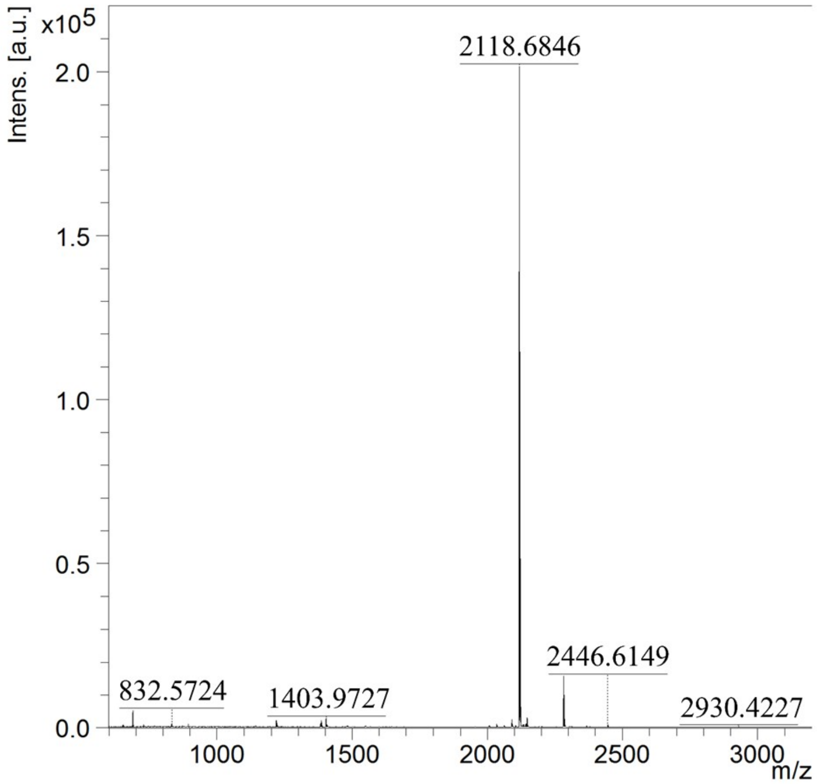

Fig. S17 HRMS spectrum of **2T**. MALDI-TOF-MS m/z: Found 2118.6846 $[M+H]^+$. Calculated For $C_{142}H_{224}N_2O_6S_2$: 2117.6726.

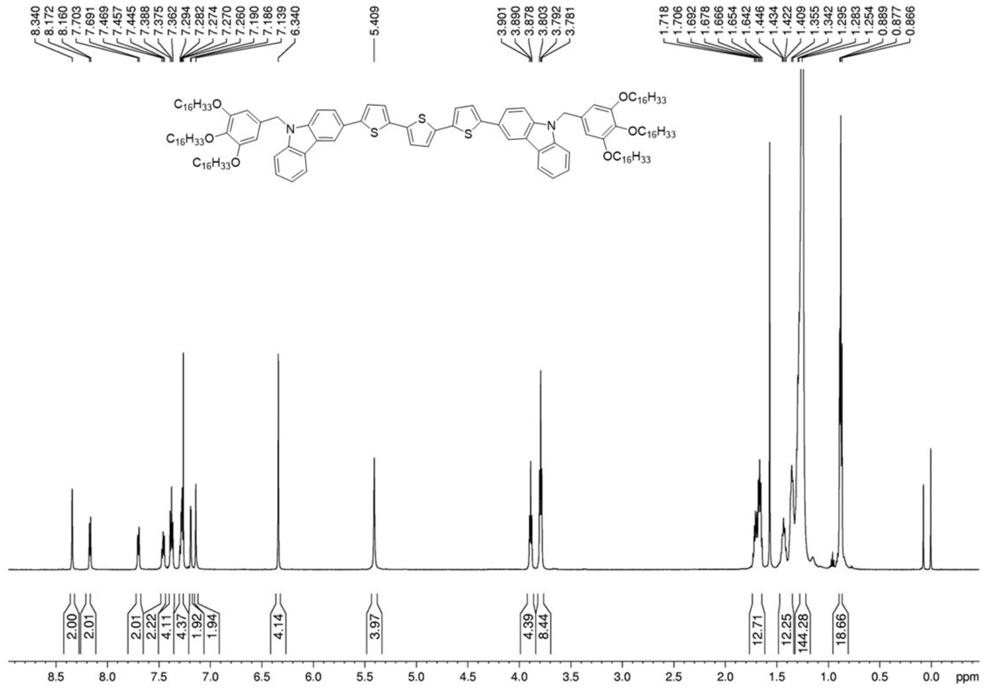

Fig. S18 ^1H NMR ($CDCl_3$, 400 MHz ppm) spectrum of **3T.**

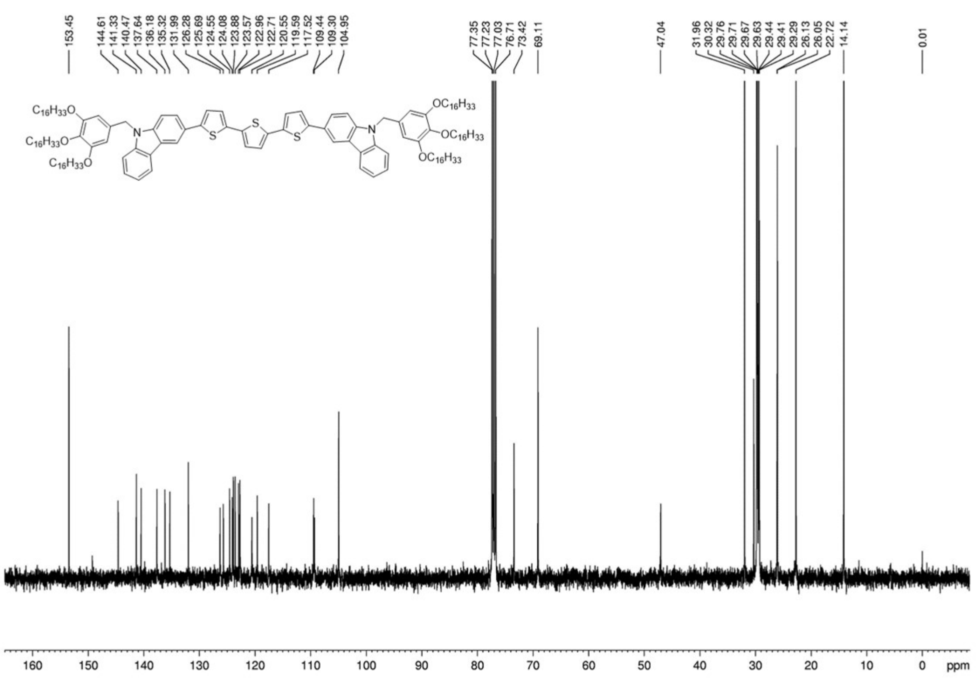

F

ig. S19 ^{13}C NMR ($CDCl_3$, 100 MHz ppm) spectrum of **3T.**

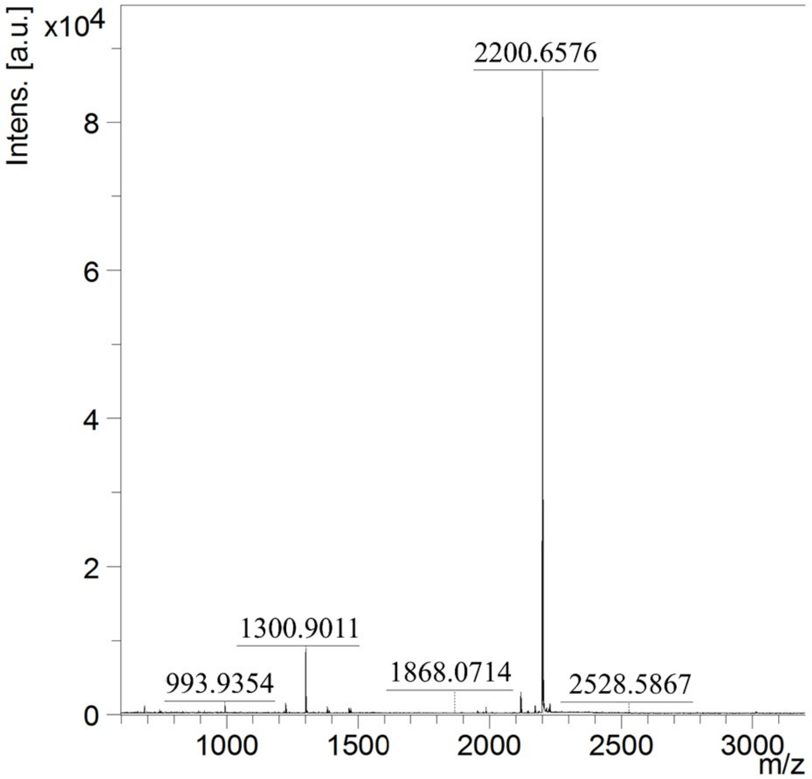

Fig. S20 HRMS spectrum of **3T**. MALDI-TOF-MS m/z: Found 2200.6576 $[M+H]^+$. Calculated For $C_{146}H_{226}N_2O_6S_3$: 2199.6603.

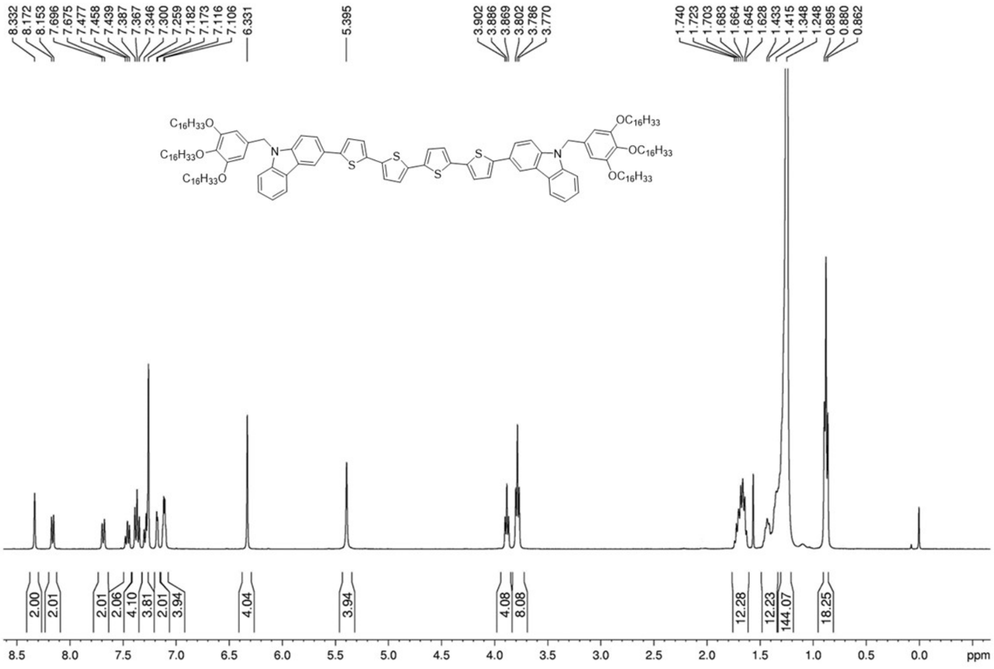

Fig. S21 ^{1}H NMR ($CDCl_3$, 400 MHz ppm) spectrum of **4T.**

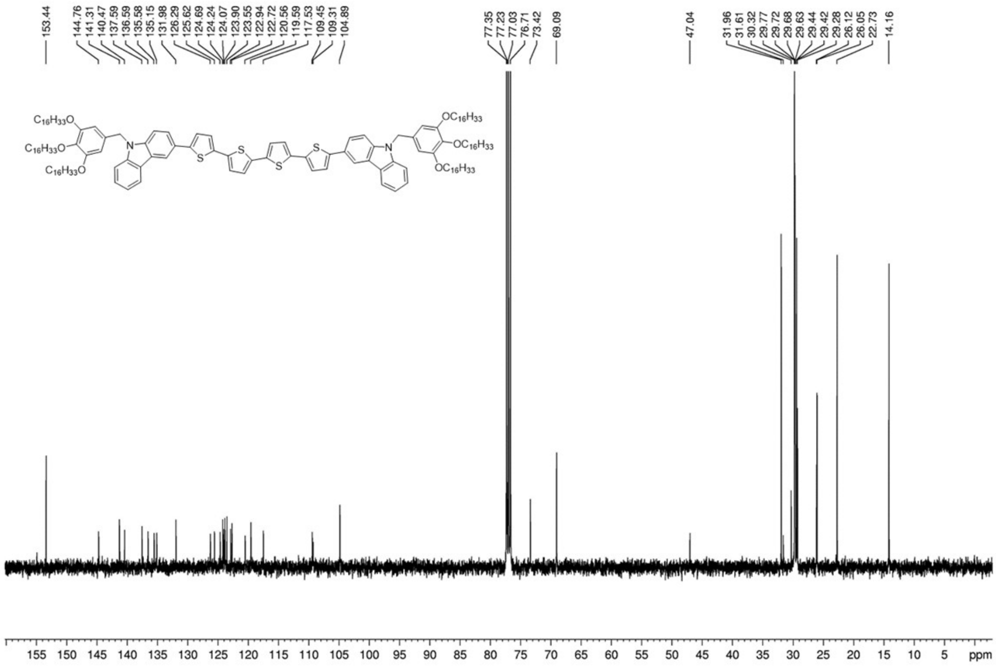

Fig. S22 ^{13}C NMR ($CDCl_3$, 100 MHz ppm) spectrum of **4T.**

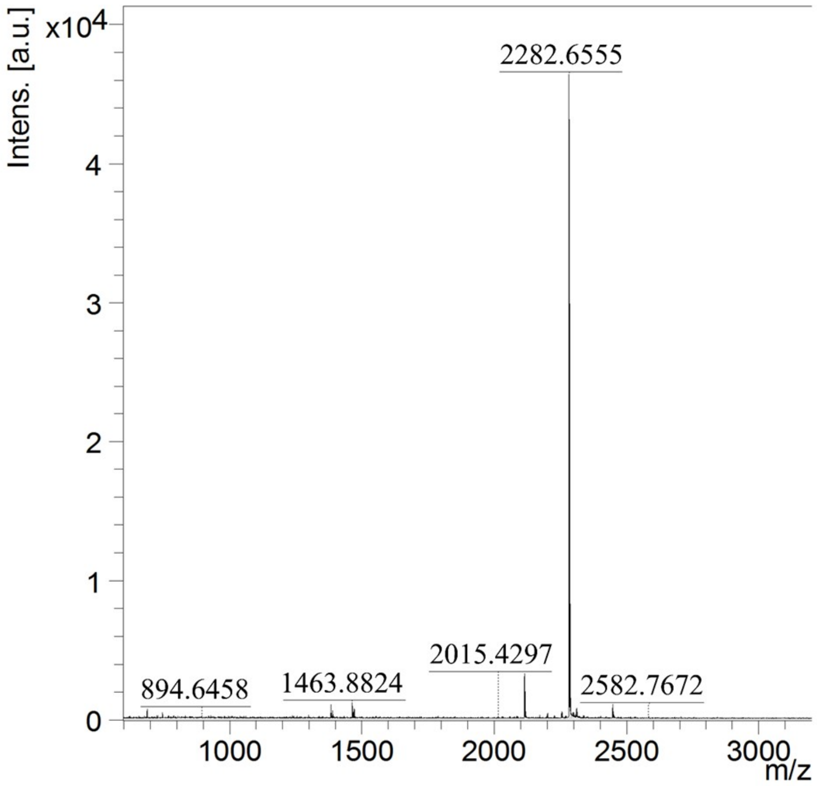

Fig. S23 HRMS spectrum of **4T**. MALDI-TOF-MS m/z: Found 2282.6555 $[M+H]^{+}$. Calculated For $C_{150}H_{228}N_2O_6S_4$: 2281.6480.

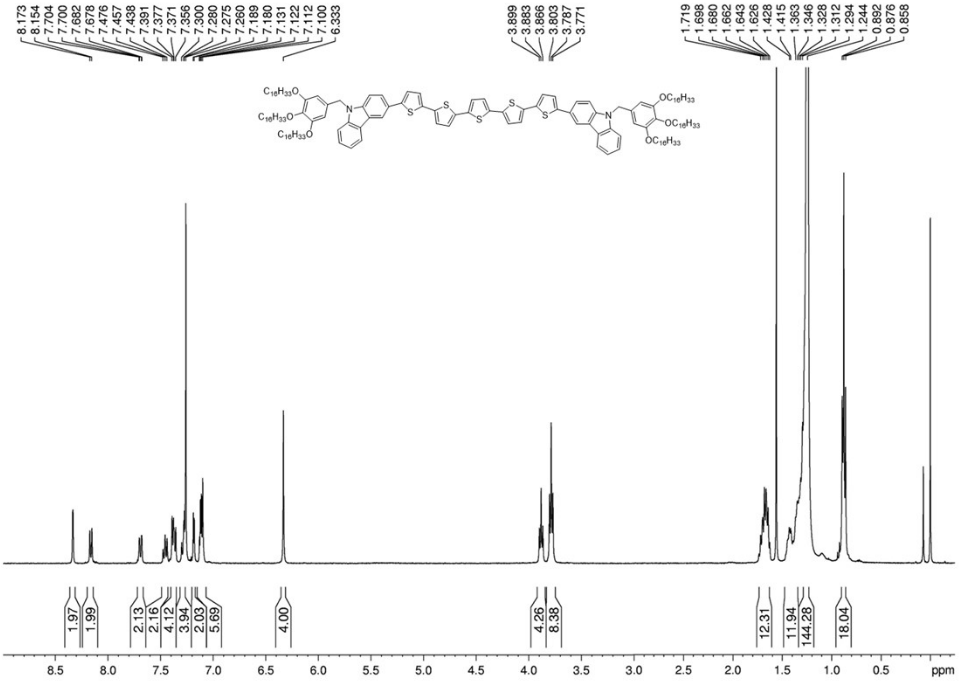

Fig. S24 ^{1}H NMR ($CDCl_3$, 400 MHz ppm) spectrum of **5T.**

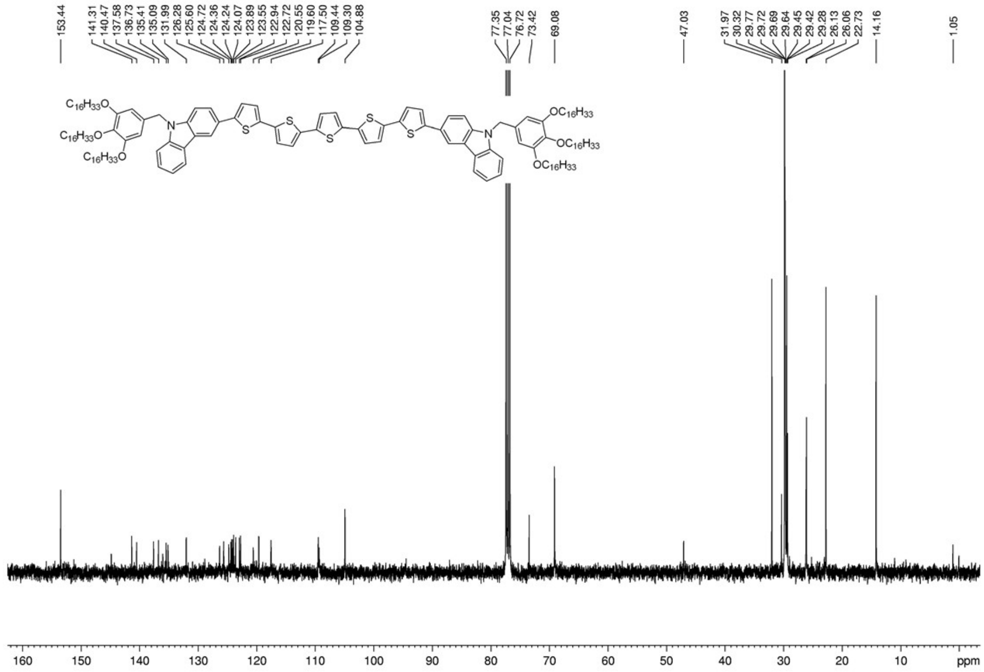

Fig. S25 ^{13}C NMR ($CDCl_3$, 100 MHz ppm) spectrum of **5T.**

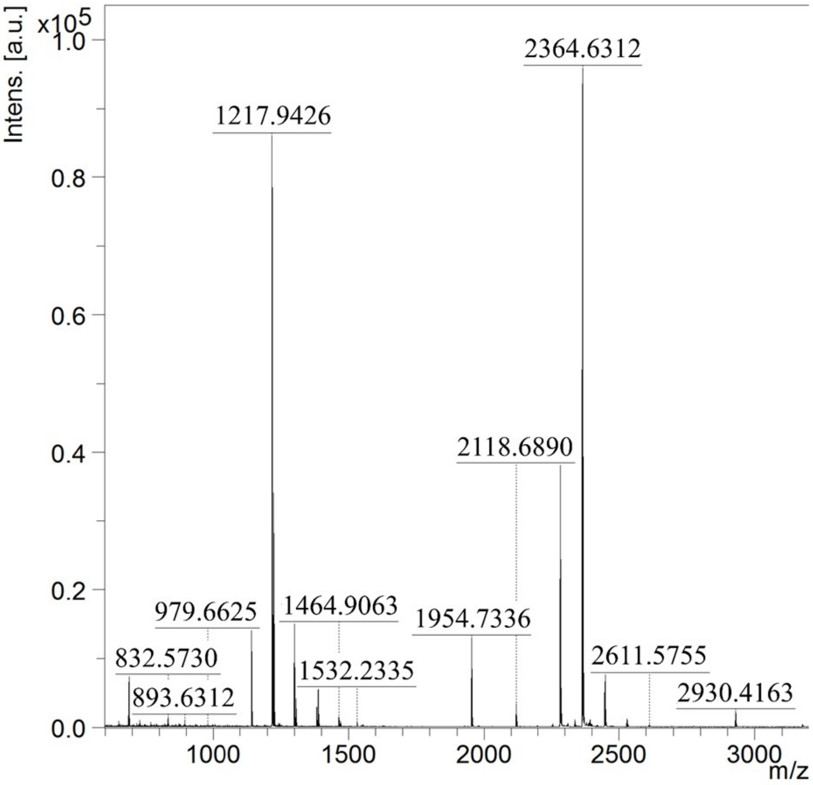

Fig. S26 HRMS spectrum of **5T**. MALDI-TOF-MS m/z: Found 2364.6312 $[M+H]^+$. Calculated For $C_{154}H_{230}N_2O_6S_5$: 2363.6357.

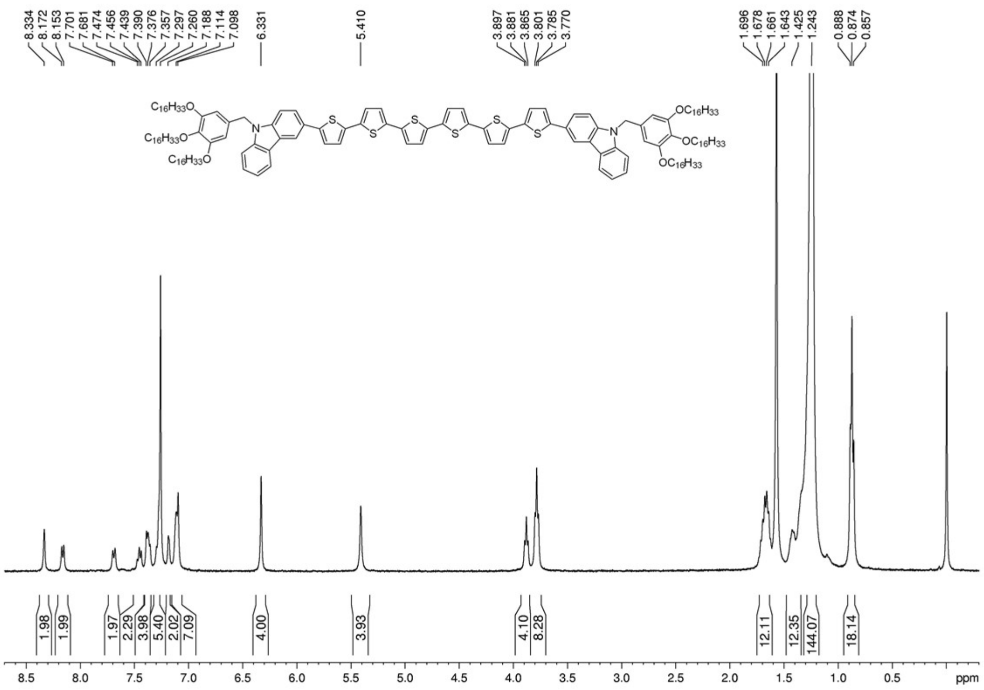

Fig. S27 ^{1}H NMR ($CDCl_3$, 400 MHz ppm) spectrum of **6T.**

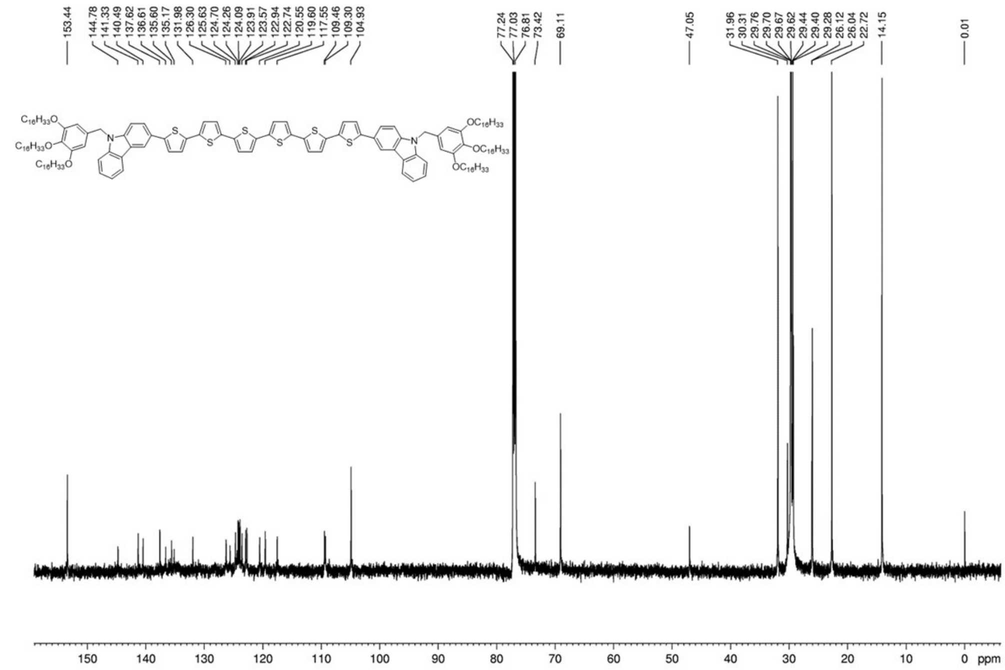

Fig. S28 ^{13}C NMR ($CDCl_3$, 100 MHz ppm) spectrum of **6T.**

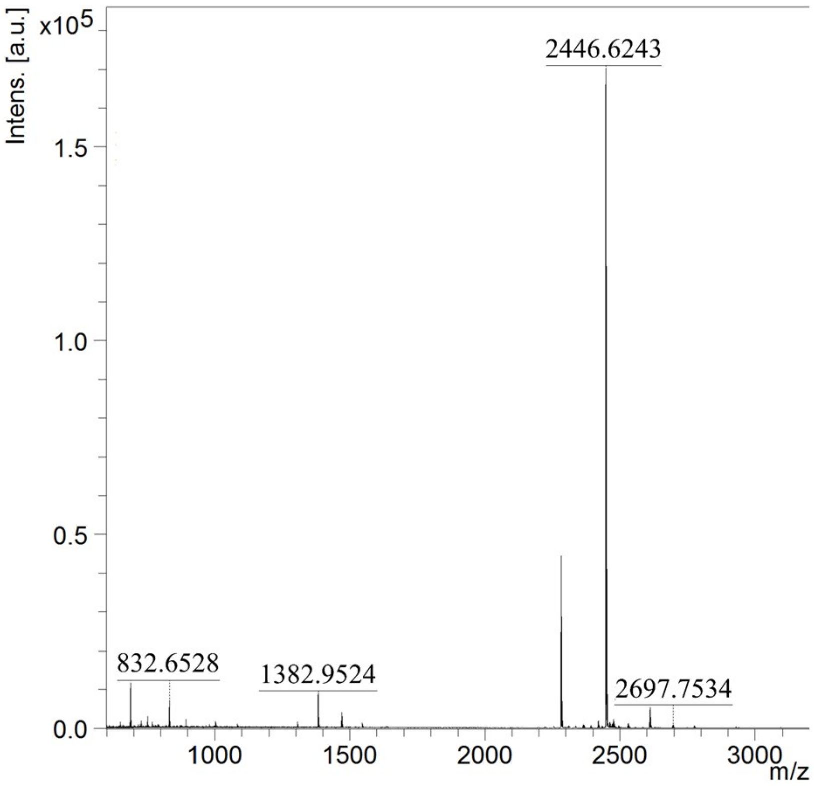

Fig. S29 HRMS spectrum of **6T** MALDI-TOF-MS m/z: Found 2446.6243 $[M+H]^+$. Calculated For $C_{158}H_{232}N_2O_6S_6$: 2445.6235.

3. References

[S1] (a) C. A. Parker and W.T. Rees, Analyst, 1960, 85, 587-600. (b) A.T. R. Williams, S. A. Winfield and J. N. Miller, *Analyst*, 1983, **108**, 1067-1071.

[S2] (a) R. De, M. Maity, A. Joseph, S. Prasad Gupta, Y. Nailwal, M. A. G. Namboothiry and S. Kumar Pal, *Small*, 2024, **20**, 2308983. (b) A. Martínez-Bueno, S. Martín, J. Ortega, C. L. Folcia, R. Termine, A. Golemme, R. Giménez and T. Sierra, *Chem. Mater.* 2024, **36**, 4343-4356. (c) H. K. Bisoyi and Q. Li, *Progress in Materials Science*, 2019 **104**, 1-52.

[S3] S. B. Chen, T. Ma, X. Y. Du, M. S. Mo, Z. L. Wang, X. H. Cheng, *J. Mol. Liq.*, 2023, **373**, 121239.
